# (Dimesityl)boron Benzodithiophenes: Synthesis, Electrochemical, Photophysical and Theoretical Characterization

**DOI:** 10.1002/open.202100265

**Published:** 2022-01-21

**Authors:** Luigi Menduti, Clara Baldoli, Serena Arnaboldi, Andreas Dreuw, Duygu Tahaoglu, Alberto Bossi, Emanuela Licandro

**Affiliations:** ^1^ Department of Chemistry University of Milan Via C. Golgi 19 20133 Milano Italy; ^2^ CNR-Institute of Chemical Sciences and Technologies (SCITEC) “Giulio Natta” and SmartMatLab Centre Via C. Golgi 19 20133 Milano Italy; ^3^ Via Fantoli 16/15 20138 Milano Italy; ^4^ Interdisciplinary Center for Scientific Computing University of Heidelberg Im Neuenheimer Feld 205 69120 Heidelberg Germany

**Keywords:** benzodithiophenes, boron, functional materials, sulfur heterocycles, triarylboranes

## Abstract

Triarylboranes containing linear or angular benzodithiophene moieties and bearing one or two dimesitylboron units were synthesized. The electrochemical and optical features of these compounds were investigated by cyclic voltammetry, UV/Vis and fluorescence spectroscopy while DFT calculations were run to analyze the energetic landscape of these systems. For both linear and angular benzodithiophenes, symmetrical disubstitution leads to the highest photoluminescence yields. The linear benzodithiophene disubstituted with two dimesitylboron units proved to be the most interesting and promising molecule as an electron‐transport material for organic electronics owing to its LUMO energy level of −2.84 eV which is close to those of commonly used electron transport materials like bathocuproine or bathophenantroline.

## Graphical abstract

**Functionalization of benzodithiophenes** with (dimesityl)boryl moieties provided derivatives with remarkable electrochemical and spectroscopic properties. The introduction of two boryl groups on the linear benzodithiophene yielded a stable blue‐emitter with high potential as electron‐transport material and naked‐eye fluorescence turn‐off sensor.

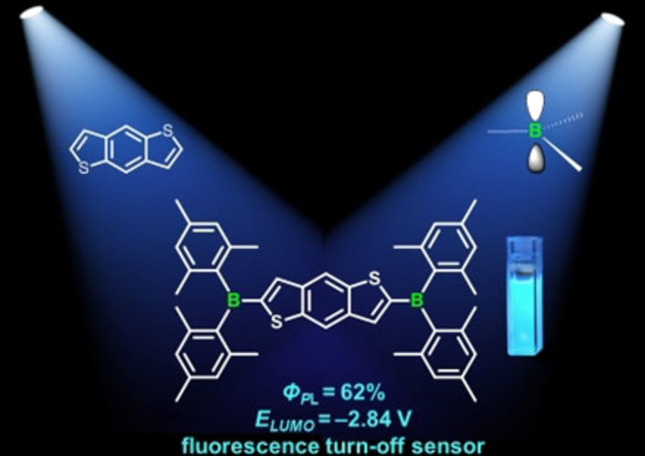

## Introduction

**Figure 1:**
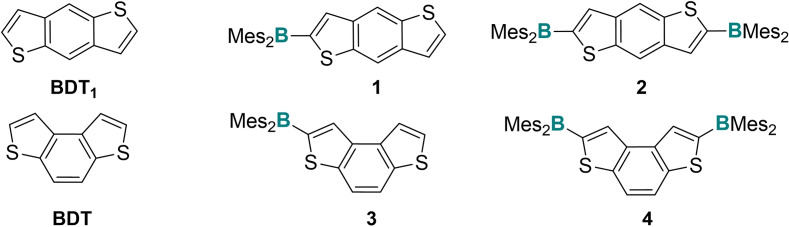
Linear (BDT_1_) and angular (BDT) benzoditiophene‐based triarylboranes.

Among thiophene‐based π‐conjugated molecules, benzo[1,2‐b:4,5‐b′]dithiophene (BDT_1_) and its angular isomer benzo[1,2‐b:4,3‐b′]dithiophene (BDT) (Figure 1) have been demonstrated to be very efficient electron‐rich building blocks in the construction of various organic material for optoelectronic and photovoltaic applications.[^1^, ^2^] The rigid and conjugated skeleton of benzodithiophene and its structural symmetry enhance electron delocalization and promote co‐facial π–π stacking in the solid state; moreover, the incorporation of substituents on the central benzene core allows the modulation of the electronic properties of such molecules and derived systems.^[3]^ Despite the extensive use of these heterocycles in the synthesis of small molecules, heterohelicenes and polymeric materials, to date, only few examples of systems containing benzoditiophene and boron such as BDT_1_‐containing BODIPY^[4]^ or bis‐carborane^[5]^ compounds are known in the literature. In contrast, no benzothiophene‐based triaryl boranes have yet been reported. Remarkable features of tri‐coordinate boron atom in arylboranes are the strong electron acceptor character due to the p‐π interactions between the empty p_z_‐orbital of boron and π‐orbitals of the conjugated frameworks, the trigonal planar geometry at the boron atom and the Lewis acid behavior. Thanks to these features, arylboron molecules have found applications in nonlinear optics,^[6]^ as two‐photon absorbing materials,^[7]^ for anion sensing^[8]^ and in OLEDs.^[9]^ The presence of electron‐donating moieties in the scaffold of arylboranes generates strong intramolecular charge transfer with significant perturbation of the optical and electronic properties. The development of novel triarylborane derivatives is therefore particularly compelling (i) in the field of electronic materials with electron transport properties due to their intrinsic deep LUMO level and hole blocking features,[^9b^, ^9f^] and (ii) as anion/Lewis base sensor materials. Many examples of thiophene‐based boranes are present in the literature,^[10]^ while only few cases of boranes containing poly‐condensed thiaheterocycles such as thienothiophene and terthienobenzene have been reported.[^11^, ^12^] To the best of our knowledge, triheteroaryl boranes derived from BDT_1_ or BDT are still unknown. Our group has a long‐standing experience in the synthesis and functionalization of BDT and BDT_1_ derivatives for obtaining more complex molecular systems.^[13]^ Given the high potential of electron‐donor containing triarylboranes for studies in material science, in this paper we describe the synthesis, electrochemical, spectroscopical and computational characterization of benzodithiophene based triheteroarylboranes **1**–**4** (Figure 1). Molecules **1** and **2** contain the linear benzodithiophene (BDT_1_) moiety and bear one or two dimesitylboron units, while the similar structures **3** and **4** incorporate the angular benzodithiophene (BDT) core.

## Results and Discussion

**Scheme 1:**
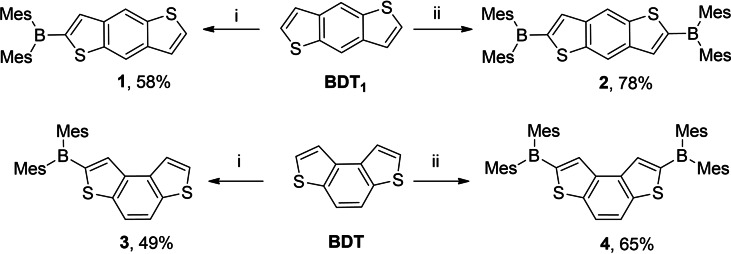
i) *n*‐BuLi (1.5 m solution, 1.2 equiv.) −78 °C, 1 h; Mes_2_BF (1.1 equiv.) ii) *n*‐BuLi (1.5 m solution, 3 equiv.) −78 to −10 °C, 1 h; Mes_2_BF (2.2 equiv.).

The synthesis of compounds **1**–**4** was carried out taking advantage of the easy and selective generation of BDT/BDT_1_ mono‐ and di‐anions through direct deprotonation of the thiophene α‐position(s) with *n*‐BuLi, followed by the addition of the corresponding boron reactant. In detail, compounds **1**–**4** (Scheme 1) were prepared reacting the parent BDT_1_ and BDT with the appropriate stoichiometric amount of *n*‐BuLi at −78 °C, followed by addition of 1 or 2 equivalents of dimesitylboron fluoride.

Due to the competitive formation of di‐substituted products **2** and **4** – the introduction of the electron attracting boryl group renders hydrogen atoms in α‐position of the second thiophene ring of **1** and **3** more acidic than those of the starting benzothiophenes BDT_1_ and BDT – mono‐substituted compounds **1** and **3** were isolated in moderate yields (58 % and 49 % respectively).

Disubstituted derivatives **2** and **4** were directly obtained in rather good yields (78 % and 65 % respectively), using 3 equivalents of *n*‐BuLi at −78 °C followed by the addition of 2.2 equiv. of Mes_2_BF.

All the obtained dimesitylboryl derivatives **1**–**4** are stable in air and in common organic solvents, and were easily purified by silica gel column chromatography and fully characterized.

### Electrochemical Characterization of Compounds 1–4

In order to obtain insights about the electrochemical features and to evaluate the role of the boron atom on the electronic properties of these new systems, the electron transfer properties of mono‐ and bis‐dimesitylboryl benzodithiophene based compounds **1**–**4**, were investigated by cyclic voltammetry (CV) and also compared with those of parent linear BDT_1_ and angular BDT.

Measurements were carried out in dichloromethane (DCM) and dimethylformamide (DMF) as solvents and tetrabutylammonium perchlorate (TBAP) as supporting electrolyte (0.1 m) in a three electrode minicell on a glassy carbon (GC) support as working electrode at 0.2 V s^−1^ potential scan rate.

**Figure 2:**
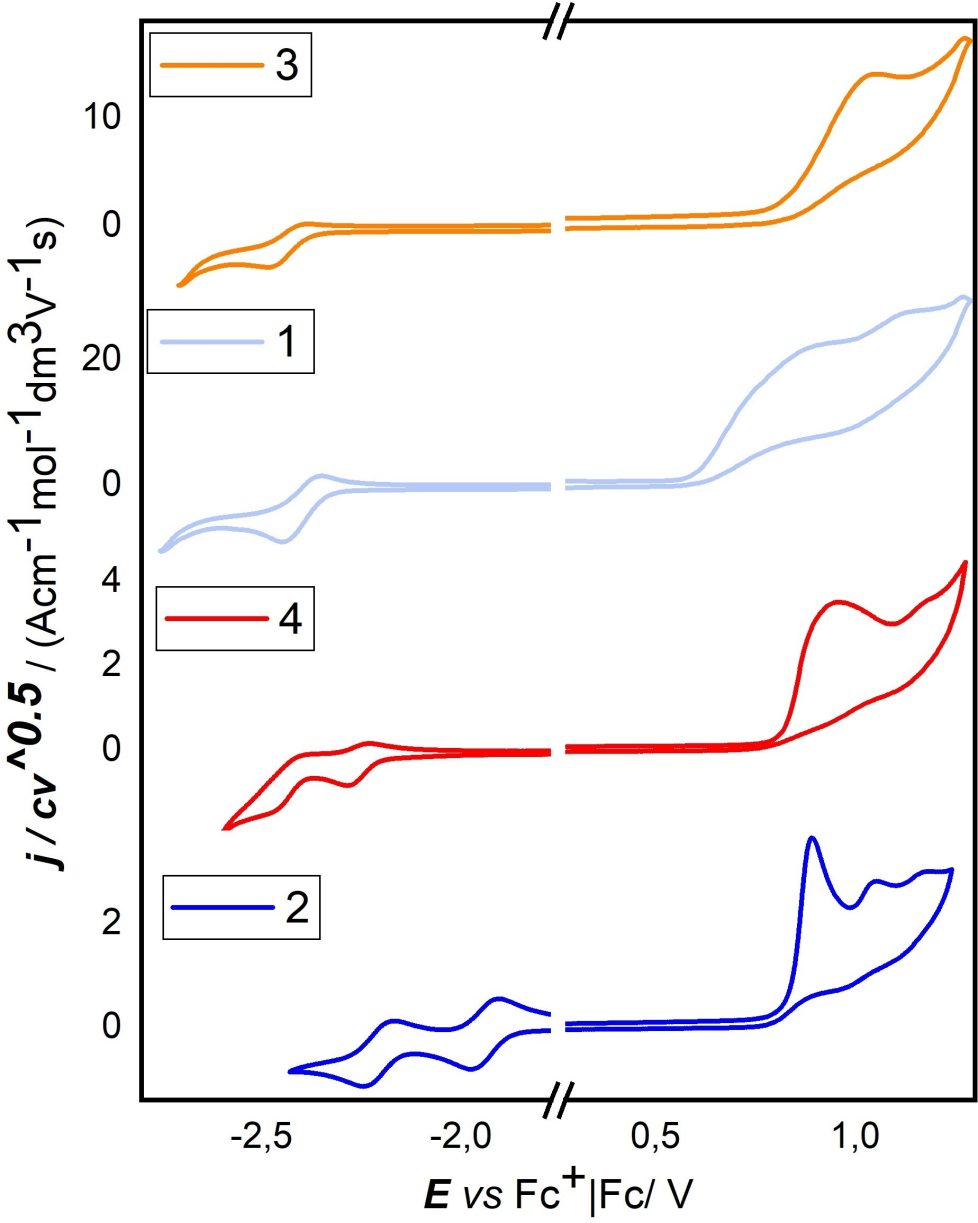
CV features of compounds **1**–**4** recorded in DCM+0.1 m TBAP at 0.2 V s^−1^ potential scan rate on a GC electrode.

A synopsis of normalized CV features for the whole series in DCM is depicted in Figure 2 and the corresponding values are collected in Table 1. The corresponding CV features in DMF are reported in the Supporting Information (Figure S3).

**Table 1:** Peak potential values for compounds **1**–**4** and parent molecules BDT1 and BDT^[13]^ with corresponding HOMO/LUMO energy levels and gaps. CVs were recorded in DCM+TBAP 0.1 m, on a GC electrode, at 0.2 V s^−1^ potential scan rate. Potential values are referred to the intersolvental redox couple [FcH]^+^ | FcH.

Compound	*E*_Ia,p_^[a]^ [V]	*E*_Ic,p_^[a]^ [V]	*E*_IIc,p_^[a]^ [V]	*E*_HOMO_^[b]^ [eV]	*E*_LUMO_^[c]^ [eV]	*E*_g_ [eV]
**1**	1.03	−2.39	//	−5.83	−2.41	3.42
**2**	0.90	−1.96	−2.23	−5.70	−2.84	2.86
**3**	1.13	−2.42	//	−5.93	−2.38	3.55
**4**	1.06	−2.12	−2.68	−5.86	−2.68	3.18
**BDT_1_**	1.01	−2.93	/	−5.81	−1.87	3.94
**BDT**	1.10	−3.00	/	−5.90	−1.80	4.10

[a] vs. [FcH]^+^ | FcH; [b] E_HOMO_=−1e×[(E_p,Ia_/V([FcH]^+^ | FcH)+4.8 V([FcH]^+^ | FcH vs. zero)] (maxima criterion); [c] E_LUMO_=−1e×[(E_p,Ic_/V([FcH]^+^ | FcH)+4.8 V([FcH]^+^ | FcH vs. zero)] (maxima criterion).

Anodic scans for all derivatives **1**–**4** (Figure 2) show one first irreversible oxidation peak corresponding to a process localized on the benzodithiophene unit; second and third peaks are related to further chemical follow‐up reactions since they disappear at increasing scan rates (Figure S4). The slightly higher oxidation potential values (Table 1) observed for angular compounds **3** and **4** (1.13 V and 1.06 V vs. [FcH]^+^ | FcH, respectively) compared to those of linear derivatives **1** and **2** (1.03 V and 0.90 V vs. [FcH]^+^ | FcH) reflect the electrochemical behavior of the parent benzodithiophenes (linear BDT_1_ shows more efficient conjugation than the angular isomer).^[14]^

Cathodic scans of the two mono‐borylated molecules **1** and **3** show, in both cases, one reversible reduction peak (−2.39 V and −2.42 V vs. [FcH]^+^ | FcH, respectively), related to the electron transfer forming the radical anion localized on the boron atom.^[15]^

The second irreversible reduction peaks, visible only for CVs recorded in DMF (Figure S3), correspond to the formation of the dianion involving a monoelectronic electron transfer and fast follow‐up reactions (formation of decomposition products).^[14]^

The two symmetric bis‐borylated molecules **2** and **4**, on the cathodic side, display two monoelectronic reversible waves due to a stepwise reduction corresponding to the two equivalent interacting redox sites localized on the boron centers, although, in the case of compound 4, the second reversible wave is partially covered by the background. From the potential values related to the first reduction and first oxidation peaks, LUMO and HOMO energies of **1**–**4** can be calculated with the maxima criterion. The resulting values, together with the corresponding energy gaps (*E_g_*), are reported in Table 1. As reported for similar compounds,^[10]^ calculated values revealed that the introduction of a trigonal boron atom to the benzodithiophene cores contributes to lowering the LUMO level without considerable effect on the HOMO. The introduction of one dimesitylboryl function on both the linear and angular benzodithiophene (compounds **1** and **3**) resulted in a ≈0.5 eV reduction of the LUMO level, while an almost doubled decrease (≈1.0 eV) was observed for the bis‐functionalized compounds **2** and **4** and, as a consequence, all molecules **1**–**4** showed narrowed energy gaps with respect to the parent benzodithiophenes. The smallest *E*_g_ values were observed for linear benzodithiophene derivatives **1** and **2**, according to the better stabilization of the localized charges achieved in the linear benzodithiophene over the angular isomer.

### Photophysical Properties

**Figure 3:**
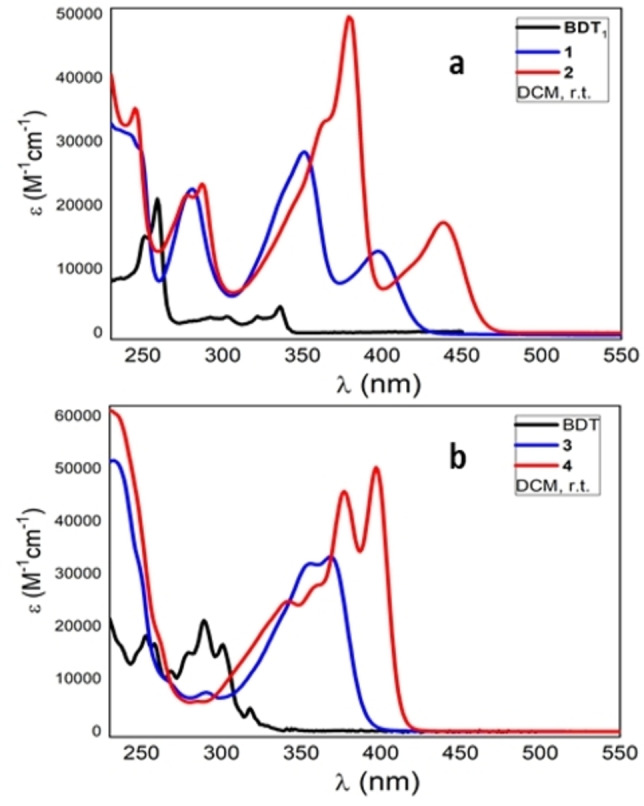
UV/Vis absorption spectra in DCM solution: a) BDT_1_ and boranes **1**, **2**; b) BDT and boranes **3**, **4**.

Compounds **1**–**4** were spectroscopically characterized in diluted DCM solution (≈10^−5^ m) at room temperature and in a glassy matrix at 77 K. The molar absorptivities of boranes **1**–**4** together with those of the parent precursors BDT_1_ and BDT are shown in Figure 3. Table 2 summarizes the main photophysical parameters of the studied compounds.

**Table 2:** Optical properties of compounds **1**–**4** in DCM at r.t. and in 2Me‐THF at 77 K.

Cmpd.	*λ_abs_* [nm] (ϵ×10^4^ [m ^−1^cm^−1^ ])	*λ_em_* [nm]	Stokes shift^[a]^ [cm^−1^]	S/T split^[b]^ [cm^−1^]	QY/(N_2_)^[c]^	τ/(N_2_)^[d]^ [ns]	k_r_ (×10^7^) [s^−1^]	k_nr_ (×10^7^) [s^−1^]	*λ_em_* ^[e]^ [nm]	Stokes shift^[a]^ [cm^−1^]	S/T split^[b]^ [cm^−1^]	τ [ns] τ^[ph]^ [ms]
**BDT_1_**	335 303 260	340	/	/	/	/	/	/	335, 486^[ph]^	/	9270	/
**BDT**	318	320	/	/	/	/	/	/	316, 456^[ph]^	/	9720	/
**1**	397 350 (2.8) 280	440	2460	/	0.21 (0.26)	2.6 (2.8)	9.3	26.4	427, 584^[ph]^	800	6300	7.2 4.4^[ph]^
**2**	436 379 (4.9) 285	469	1630	/	0.56 (0.62)	5.8 (6.1)	10.2	6.2	461	430		7.31
**3**	369 (3.3)	410 555^[ph]^	2710	6370	0.03 (0.03)	0.136	24.2	7.1	397, 543^[ph]^	790	6770	0.6 45.8^[ph]^
**4**	397 (5.0) 359	411, 587^[ph]^	860	7300	0.05 (0.05)	0.129	41.1	7.3	410, 573^[ph]^	480	6940	0.154 and 1.05 22.6^[ph]^

[a] Measured at the peak maxima; [b] energy difference between the singlet and triplet emissive states; [c] absolute quantum yield (QY) in air‐saturated solutions (QY measured in N_2_‐saturated solution); [d] lifetime in air‐saturated solutions (lifetime in N_2_‐saturated solution);[e] emission wavelength in a frozen 2‐methyl‐THF solution (77 K); [ph] phosphorescence. Photoluminescence measurements were carried out in diluted solutions corresponding to an optical density below 0.1 at the wavelength used for excitation.

In particular, parent BDT_1_ and BDT show well‐defined absorptions (Figures 3a, 3b – black lines), characterized by a significant vibronic structure, as typically observed for rigid and conjugated polycyclic systems. The introduction of one dimesitylboryl group, as in **1** and **3** (Figures 3a, 3b – blue lines), results in a significant bathochromic spectral shift (≈60 nm) and a substantial change in the absorptions (band shape and intensity), owing to both the asymmetric substitution pattern of the cores and the effective conjugation between BDTs and the B(Mes)_2_ group.

Compound 1 is characterized by three major absorption bands at 400 (onset 430), 350 and 270 nm, respectively, while compound 3 is characterized by a peak at 365 nm (onset 400 nm) with some vibronic features and a second intense transition below 280 nm. Compounds **2** and **4**, containing two B(Mes)_2_ groups, display a more pronounced bathochromic shift (red lines) with respect to the parent compounds (≈100 nm), again owing to the effective conjugation over the whole molecule.

In terms of absorption shapes, mono‐ and bis‐substituted compounds show a remarkably similar feature with a red shift of 40 nm between **1** and **2** and 30 nm between **3** and **4**. In both bis‐borylated **2** and **4**, almost all transitions are more resolved due to the symmetric substitution pattern. In the latter case, two sets of transitions (330‐360 nm and 370–410 nm) blend into each other as indeed evidenced by the theoretical calculations (see below). Solvatochromic experiments were also performed on compounds **1**–**4** to study the molecular electronic transition state polarity character; the normalized spectra in solvents with increasing polarity (polarity scale *n*‐hexane <DCM<THF<CH_3_CN) are shown in Figure S1 in the Supporting Information.

Overall, the four boranes do not display appreciable solvatochromism, although, by comparing the *n*‐hexane/CH_3_CN spectra, it can be inferred that ground states are, in all cases, slightly more polar than the excited ones.

**Figure 4:**
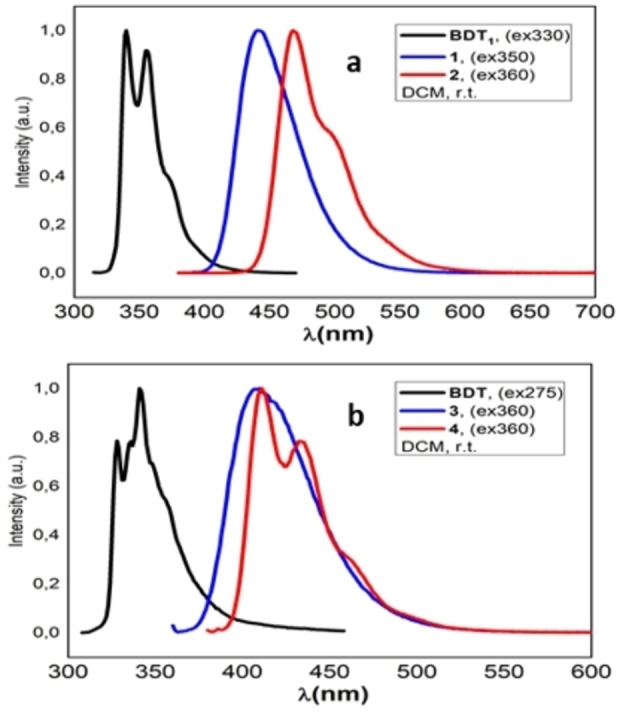
Normalized emission spectra in DCM: a) BDT_1_ and boranes **1**, **2**, b) BDT and boranes **3**, **4**.

The emission properties of boranes **1**–**4** were studied at room temperature in diluted DCM solutions and the respective spectra are shown in Figure 4 together with those of the parent BDT_1_ and BDT.

As already observed in the UV/Vis section, the introduction of one or two B(Mes)_2_ substituents to the BDT_1_/BDT cores results in a significant bathochromic shift of the emissions. Monoborylated derivatives **1** and **3** show a pseudo‐Gaussian emission band centered at 440 and 410 nm, respectively (Table 2). The corresponding bis‐functionalized compounds **2** and **4** emit at 469 and 411 nm, respectively, and their spectra are vibronically resolved as the consequence of the molecular symmetry.

The calculated Stokes shift are as large as 2500–2700 cm^−1^ in the monosubstituted systems 1 and 3, while it reduces significantly in 2 and 4 (ca. 1600–900 cm^−1^). Interestingly, the borane derivatives of linear BDT_1_ 1 and 2 show significantly higher photoluminescence efficiency compared to those of BDT as well as showing absolute fluorescence quantum yields (QY) (measured in air‐saturated solution) equal to 0.21 and 0.56, respectively, and fluorescence lifetimes of 2.6 and 5.8 ns.

On the other hand, mono‐ and bis‐borylated BDT derivatives **3** and **4** resulted to be less efficient emitters, characterized by QYs of 0.026 and 0.049, respectively, and fluorescence lifetimes of only few hundreds of picoseconds (Table 2).

**Figure 5:**
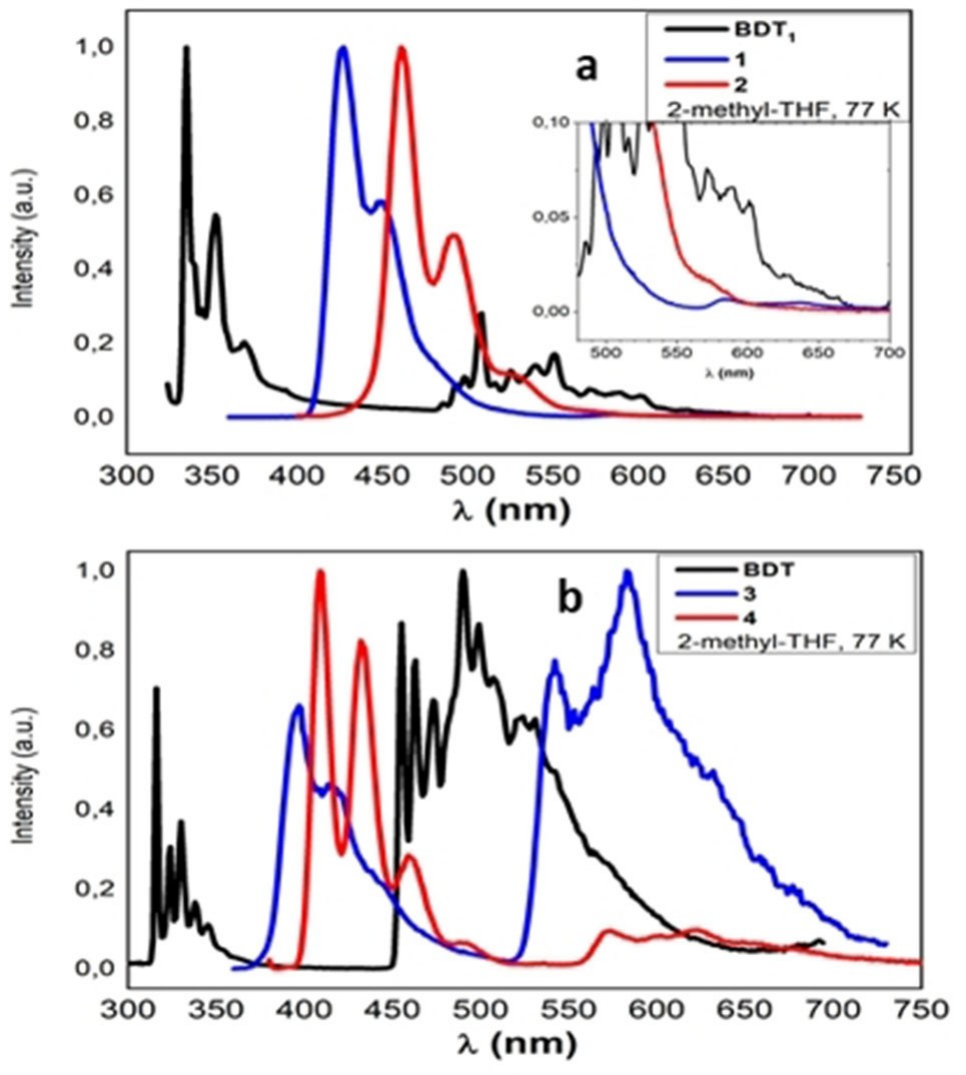
Normalized emission spectra in 2‐methyl‐THF at 77 K: a) BDT_1_ and boranes **1**, **2**; b) BDT and boranes **3**, **4**.

In order to investigate the origin of this significantly different behavior, photoluminescence experiments were repeated in oxygen free N_2_‐saturated solutions at room temperature (Figure S2) and in a frozen 2‐methyl‐THF solution at 77 K (Figure 5, Table 2).In de‐aerated solution, no appreciable differences in the emission line shape of compounds **1** and **2** are detectable, although the photoluminescence quantum yield appear slightly enhanced as reported in Table 2a. For compounds **3** and **4** a weak emission band above 550 nm interestingly appeared, which, considering the absence of oxygen quenching effect, can be attributed to the phosphorescence emission of the molecules (confirmed by low temperature experiments; see below).

In a frozen 2‐methyl‐THF solution at 77 K, the fluorescence emissions of all compounds look more vibrationally resolved and slightly hypsochromically shifted (Figure 5) compared to those recorded at room temperature; a larger shift is measured for the monosubstituted (**1**, **3**) compared to the disubstituted derivatives (**2**, **4**). Intense phosphorescence emissions (λ >500 nm) are observed for the BDT derivatives **3** and **4**, whereas only a barely perceivable phosphorescence band is observed for compound **1** (inset of Figure 5a; ≈590 nm) while, possibly hidden under the fluorescence band, such emission cannot be observed for **2**.

In all cases, the singlet triplet splitting is in the range of 6300–7000 cm^−1^. Typical organic phosphorescence lifetimes over tens of milliseconds characterize these bands as reported in Table 2. Considering the strong phosphorescence emission intensity of the BDT derivative (even at room temperature), it is possible to assume that the origin of the low quantum yield of fluorescence observed in boranes **3** and **4** can be reasonably attributable to a more efficient intersystem crossing into the triplet state of BDT. Overall, the triplet energies, calculated from the phosphorescent signals, are in the range of 2.28 and 2.16 eV for **3** and **4**, respectively, and 2.12 eV for **1**.

### Fluoride‐Sensing Properties

Owing to their intrinsic Lewis‐acidic character, triarylboranes easily form complexes with Lewis bases, but sterically hindered boranes have been reported to be sensitive only to small anions such as fluoride.^[8]^ This is the base for their use as selective sensors because the coordination of the trigonal boron atom to F^−^ generally interrupts the π‐conjugation resulting in a substantial change of the absorption/emission properties of the molecule.[^16^, ^17^] Because of their more extended conjugation and high photoluminescence performances, we considered it of particular interest to investigate the fluoride‐binding properties of the mono‐ and bis‐borylated linear BDT_1_ derivatives **1** and **2** and to this aim, UV/Vis absorption and fluorescence titration experiments were performed in THF, using tetrabutylammonium fluoride (TBAF) as the fluoride source.

#### Absorption Experiments

Absorption titration experiments of compounds **1** and **2**, conducted in THF solution using 0.1 m TBAF as fluoride source, revealed a single and a two‐step binding process, respectively.

**Figure 6:**
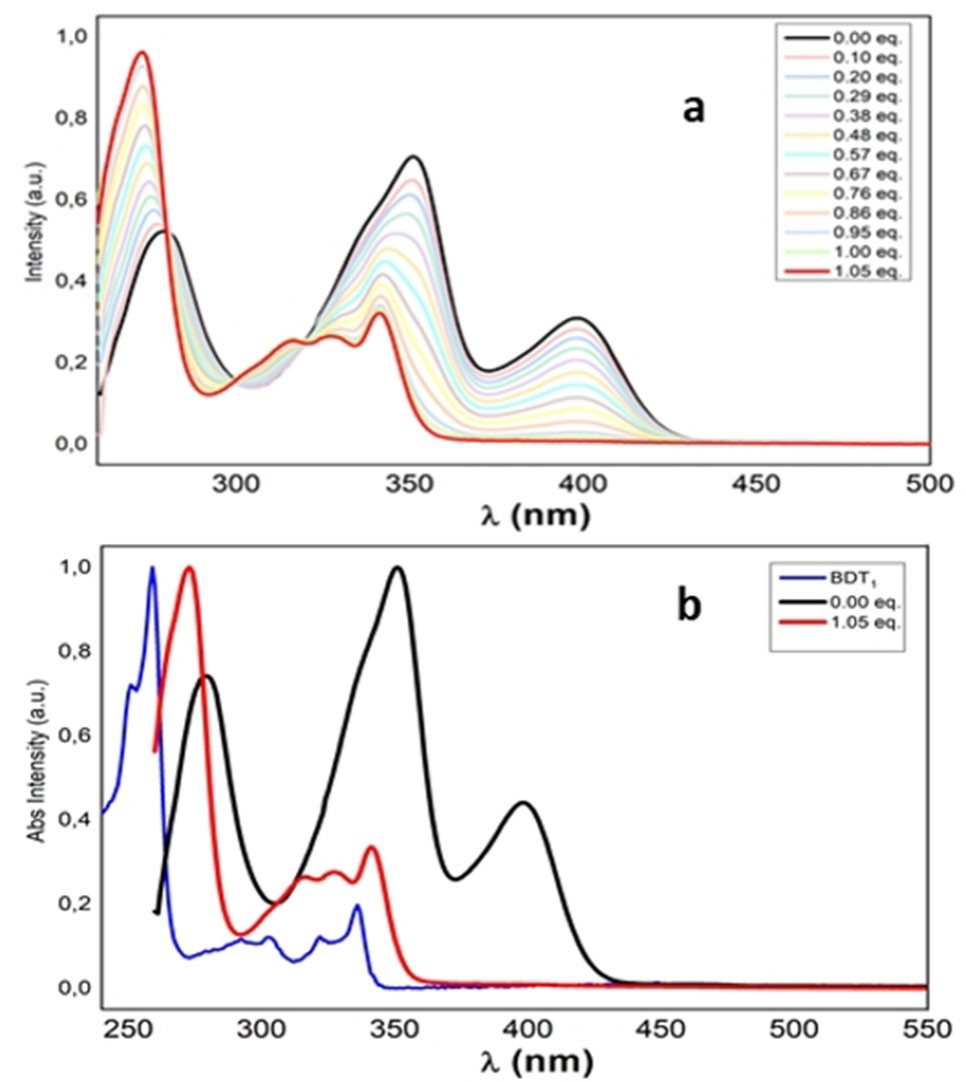
a) UV/Vis titration experiment in THF of **1** (2.12**×**10^−5^ m in THF) with F^−^ (0.1 m TBAF in THF); b) comparison of the absorption spectra of BDT_1_ (blue line), **1** (black line) and **1F^−^** (red line).

In detail, the treatment of monoboryl compound **1** with increasing amounts of TBAF (up to 1.05 equiv.) leads to the complete disappearance of the absorption band at 400 nm and a progressive decrease in the intensity of the main absorption band (350 nm) accompanied by a slight blue shift (≈10 nm). The fluoride binding can be observed by naked eye as a change in the color of the solution from yellow to colorless. The appearance of two shoulders 315 nm and the isosbestic points (280, 300, 320 nm) in this concentration range can be ascribed to the formation of the fluoride‐coordinated compound **1F^−^** and the coexistence of the **1** and **1F^−^** species in solution (Figure 6). Interestingly, the absorption profile of **1** after the addition of 1.05 equiv. of fluoride resembles the absorption bands of the parent BDT_1_, thus confirming interrupted conjugation due to fluoride binding to the boron center (Figure 6b).

**Figure 7:**
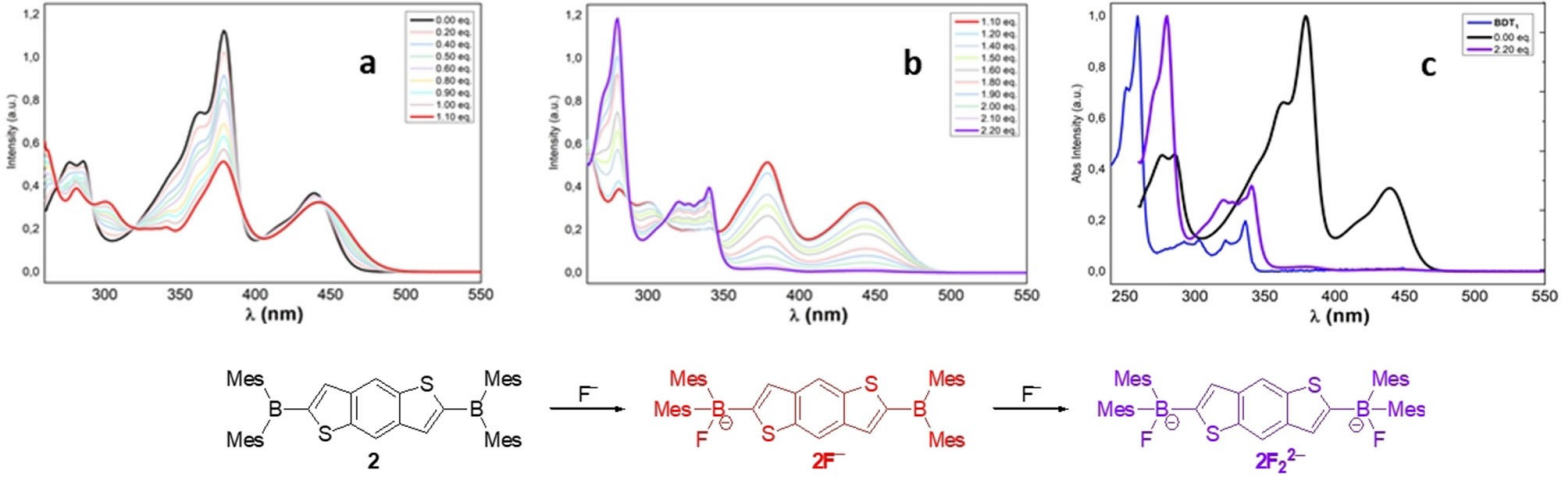
UV/Vis titration experiment of 2 (1.89×10^−5^ m in THF) with F^−^ (0.1 m TBAF in THF): a) fluoride concentration range: 0.00–1.10 equivalents; b) fluoride concentration range 1.10–2.20 equivalents; c) comparison of the absorption spectra of BDT_1_, 2, 2F_2_^2−^ (THF).

Absorption titration experiments of **2** with F^−^ (Figure 7) showed a two‐step binding process, in accordance with the presence of two boron centers. In detail, the treatment of **2** with 1.10 equiv. showed a decrease of the absorption bands at 270 nm and 380 nm and the appearance of a new band around 300 nm (Figure 7a). Titration gave rise to several isosbestic points (268 nm, 290 nm, 320 nm, 391 nm, 403 nm, 445 nm) which can be attributed to the formation of the species **2F** ^−^ and the concomitant presence of **2** in solution. Progressive addition of fluoride up to 2.20 equiv. led to the disappearance of the bands at 378 and 442 nm with concomitant increase in the intensity of the bands below 350 nm (Figure 7b). New isosbestic points (287 nm, 310 nm, 345 nm) were observed, indicating the formation of the bis‐fluorinated species **2F_2_** ^**2**−^. Similar to the observations for compound **1**, the absorption spectrum of **2** showed a “BDT_1_‐like” absorption profile after the addition of 2.20 equiv. of fluorides, thus confirming interrupted conjugation caused by complexation of the two boron centers (Figure 7c). As observed for compound **1**, spectral changes originated by the progressive addition of fluorides were accompanied by a change in the solution's color from bright yellow to colorless (upon addition of 2.4 equiv. F^−^).

### Fluorescence Experiments

**Figure 8:**
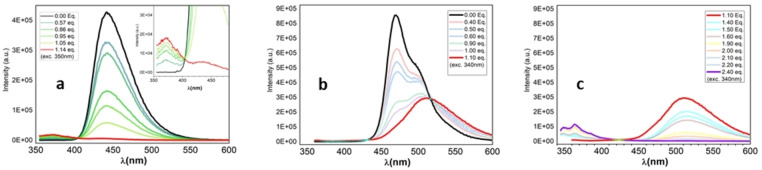
Fluorescence titration experiment of 1 and 2 with F^−^ (0.1 m in TBAF THF): a) titration of 1 (1.89×10^−5^ m in THF, exc. 350 nm); b,c) titration of 2 (2.12×10^−5^ m in THF, exc. 340 nm), fluoride concentration range 0.00–1.10 equivalents (b) and 1.10–2.40 equivalents (c).

Fluorescence titration experiments were also run in parallel to the absorption measurements. The treatment of **1** with 1.14 equiv. of F^−^ resulted in a progressive decrease in the intensity of the main emission band (440 nm) until almost complete quenching of the fluorescence. These results confirmed the formation of the monoanionic species **1F^−^** consistent with the 1 : 1 coordination of the empty pπ orbital of the boron center (Figure 8a).

**Figure 9:**
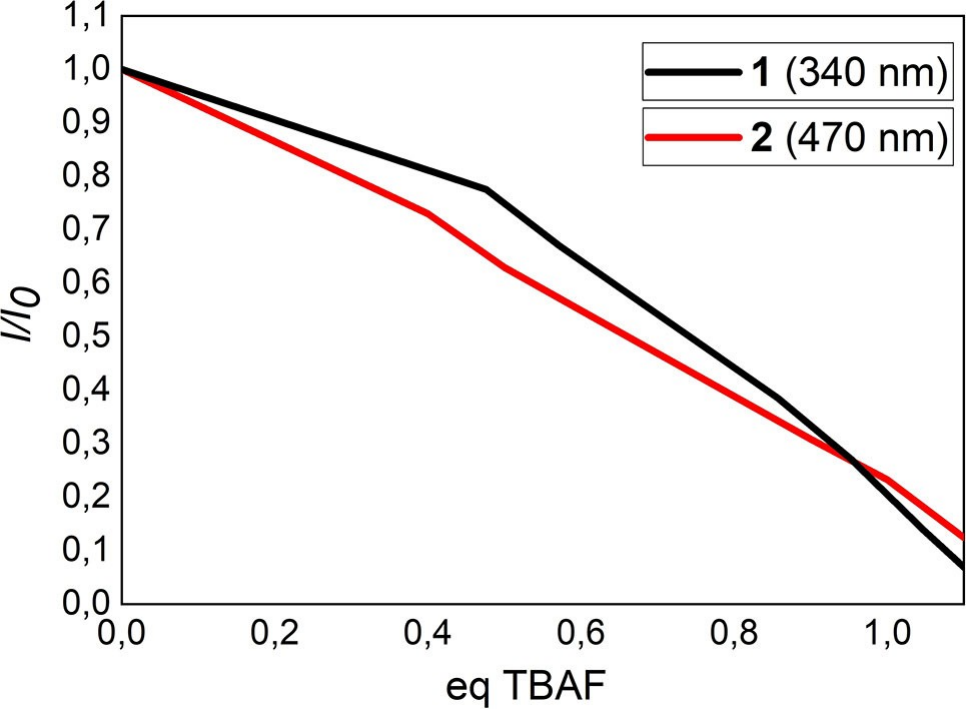
Stern‐Volmer plots for the titration of **1** and **2** by fluoride ions.

The treatment of **2** with TBAF, as expected, resulted in a two‐step fluorescence quenching process (Figure 8b, c). The addition of fluoride ions up to 1.10 equiv. resulted in a progressive intensity decrease of the main emission band (470 nm) and the appearance of a red‐shifted band at 510 nm, thus originating an isosbestic point at 516 nm which can be attributed to the formation of the monoanionic species **2F_2_** ^**2**−^ (Figure 8b). Moreover, similarly findings for comparable systems,^[18]^ the strong redshift (≈40 nm) and the broadened shape of the band at 516 nm suggest an intramolecular charge transfer process from the anionic boron center to other neutral trigonal boron atom, in accordance with the effective conjugation of the system. Further addition of fluoride up to 2.40 equiv. resulted in a decrease in the intensity of the band at 516 nm up to almost complete quenching of the fluorescence and the appearance of a new blue‐shifted band at 365 nm (Figure 8c). A new isosbestic point (around 430 nm) was observed, indicating the formation of the dianionic species **2F_2_** ^**2−**^. We next sought to evaluate the quenching efficiency of the BDT_1_ derivatives and thus prepared a Stern‐Volmer plot for compounds **1** and **2** (Figure 9). The Stern‐Volmer Plot revealed that the addition of a slight excess of fluorides (1.05–1.1 equiv.) leads to almost complete quenching (≈90 %) of the initial emission peaks (340 nm and 470 nm, respectively) for both BDT_1_ derivatives. Absorption and fluorescence titration experiments revealed that both mono‐ and bis‐borylated benzodithiophenes have a great response to the addition of fluorides, according to what had been reported for similar dimesitylboryl‐functionalized scaffolds.[^11b^, ^19^] These results indicate that compound **1** and **2** are promising scaffolds for applications as building blocks for fluoride‐detecting turn‐off sensor materials.

### DFT Calculations

**Figure 10:**
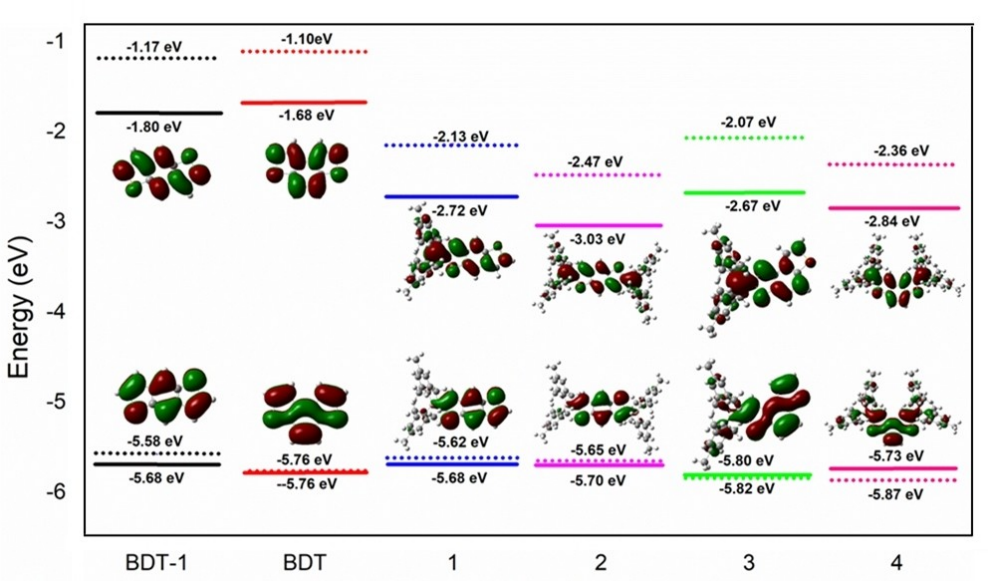
Calculated (dots; B3LYP−D3/6‐31G** in DMF(CPCM)) and experimental (solid lines, electrochemistry) HOMO and LUMO energy levels and pictorial representations of the frontier orbitals for compounds **1**–**4** and parent BDT_1_ and BDT. The isovalue is consistently set to 0.05.

DFT calculations were performed on boron‐compounds **1**–**4** (Figure 10) to further explore their properties. Geometries were optimized at the B3LYP/6‐31g** level of theory^[20]^ with Grimme's D3 dispersion model.^[21]^ The CPCM[^22^, ^23^] polarizable conductor calculation model was used as a solvation method and dimethylformamide (DMF) was used as solvent. All ground state calculations were carried out using the Gaussian09 program package.^[24]^

DFT calculations reveal that the functionalization with boryl groups narrows down the energy gap between the frontier orbitals by decreasing the LUMO energy levels by ≈1.0 eV and ≈1.3 eV for the mono‐ and bis‐borylated molecules, respectively, while the HOMO energies remain constant. Moreover, according to the analysis of the orbital distributions, there is a significant contribution of boron atoms to the LUMO wave function density as it is clearly seen from the pictorial frontier orbitals (Figure 10).

In the borylated molecules, HOMOs are mostly delocalized on the benzodithiophene fragments while the LUMOs extend over the boryl group with the contribution of boron atoms in π‐conjugation and are mainly localized on the boron atom (Figure S5).

Calculated HOMO and LUMO energies generally strongly depend on the chosen calculation level and environment model, therefore they cannot be quantitatively compared with experimental CV values. Our computed LUMO energy levels are consistently overestimated compared to the experimental values from the electrochemical measurements; however, the observed trends are in very good agreement.

Vertical and adiabatic electron detachment and attachment energies are given in Table 3. Positive detachment energies show that neutral molecules have lower energy than the cations, so energy is required to remove an electron from the molecules. Negative attachment energies mean that molecules are likely to accept electrons and have lower energy in their anionic form.

**Table 3:** Calculated vertical and adiabatic detachment energies (VDE, ADE) and vertical and adiabatic electron attachment energies (VAE and AAE) using B3LYP/6‐31G** in DMF (the values in parenthesis were calculated using B3LYP/6‐31++G**).

	**BDT‐1**	**BDT**	**1**	**2**	**3**	**4**
VDE (eV)	5.51	5.71	5.54	5.55	5.74	5.77
ADE (eV)	5.43	5.57	5.45	5.46	5.59	5.63
VAE (eV)	−1.27 (−1.59)	−1.19 (−1.52)	−2.26 (−2.50)	−2.59 (−2.81)	−2.20 (−2.45)	−2.49 (−2.70)
AAE (eV)	−1.41 (−1.74)	−1.34 (−1.68)	−2.39 (−2.63)	−2.74 (−2.96)	−2.36 (−2.59)	−2.63 (−2.84)

Attachment energies increase when the electron deficient boryl groups are introduced to the parent molecules. Since the ionization potentials are associated with relatively constant HOMO energies, there is no significant change in these energy values.

**Figure 11:**
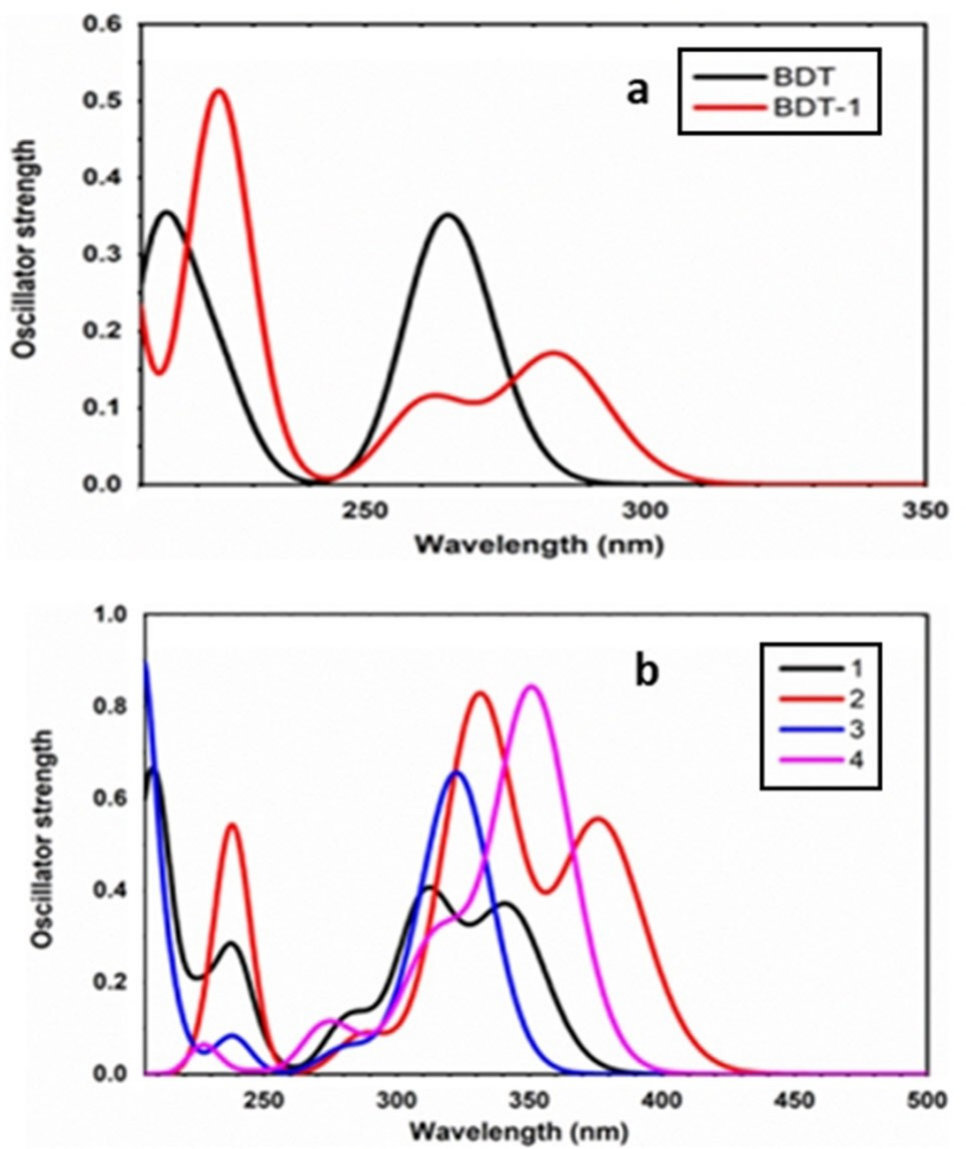
Calculated absorption spectra: a) parent BDT_1_ and BDT molecules; b) borylated structures **1**–**4**, using CAM‐B3LYP/6‐31G* level.

Although there is a shift of approximately 50 nm between the calculated and experimental peak values, the trends for the calculated absorption bands are in a good agreement with those observed in the experimental spectra (Figure 11, Table 4). According to the excited state calculations, the lowest absorption band corresponds to the electron excitation from HOMO to LUMO and a shift of electron density from the electron‐donating system to the electron‐accepting boron moiety is apparent, which is visible in the attachment/detachment densities of the first excited state of **1**–**4** (Figure S5).

**Table 4:** Wavelengths [nm] of the first absorption peak of experimental and calculated spectra.

	**BDT_1_**	**BDT**	**1**	**2**	**3**	**4**
Experimental	335	318	398	438	370	398
TD‐DFT	284	267	343	377	324	352

## Conclusion

In this paper, we have described the synthesis and characterization of four new benzodithiophene‐based triarylboranes **1**–**4** incorporating the linear (BDT_1_) or angular (BDT) benzodithiophene moieties and bearing one or two dimesitylboron units. The electrochemical and optical features of compounds **1**–**4** were investigated by cyclic voltammetry, UV/Vis and fluorescence spectroscopy while DFT calculations were run to analyze the energetic landscape of these systems.

The introduction of (Mes)_2_B groups on the BDTs systems results in a progressive shift of the absorptions toward the visible region with the presence of an intense band between 400 and 480 nm in the case of **2**. From our results, it seems that a symmetrical substitution pattern such as in compounds **2** and **4** on the conjugate benzodithiophene moiety leads to the highest photoluminescence yields compared to the monosubstituted derivatives **1** and **3**. Overall, among the studied boranes, we found compound **2** to be the most interesting and promising as an electron‐transport molecule for organic electronics, owing to its LUMO energy level of −2.84 eV which is close to those of commonly used electron transport materials like bathocuproine or bathophenantroline. Moreover, **2** proved to be a valuable sensing material owing to both the full luminescence quench upon interaction with F^−^ (turn‐off fluorescence sensing) and the visible color fading following the fluoride interaction (naked‐eye direct indicator).

## Experimental Section

**General**: If not specified, all the reactions were performed under N_2_ inert atmosphere using standard Schlenk techniques. Tetrahydrofuran was dried over Na/benzophenone and degassed prior to use. All reagents were used as received from commercial sources without further purification. Column chromatography was performed using silica gel (70–230 mesh). Melting points were determined with Stuart Scientific melting point apparatus and are uncorrected. ^1^H and ^13^C NMR spectra were recorded on Bruker AMX 300 and Bruker DRX‐400 spectrometers; chemical shifts (*δ*) are reported in parts per million (ppm), and the coupling constants (*J*) are given in hertz (Hz). High‐resolution EI mass spectra were recorded on a Vg Autospect M246. Dimesitylboron fluoride and benzo[1,2‐b:4,5‐b′]dithiophene (BDT_1_) are commercially available, while benzo[1,2‐b:4,3‐b′]dithiophene (BDT) was synthesized according to the literature.^[25]^

The absolute photoluminescence quantum yields were measured with a spectrofluorimeter Hamamatsu QY‐C11347 Quantaurus provided with a 150 W Xenon lamp, integration sphere and a multichannel analyzer. UV/Vis spectra and solvatochromic studies were performed on a Agilent 8453 PDA spectrophotometer; emission and excitation spectra were recorded with a FLS 980 (Edinburgh Instrument Ltd.). equipped with a 450 W Xenon arc lamp. The photoluminescence lifetime measurements were determined through the TCSPC (time‐correlated single photon counting) routine, using a pulsed LED source and a laser one form Edinburgh (Edinburgh Instrument Ltd.). The photoluminescence experiment at room temperature were performed in air‐saturated and nitrogen‐saturated DCM solutions in concentration of 2**×**10^−5^ m. Low temperature experiments were run in 2MeTHF solution at 77 K.

**Electrochemistry**: compounds **1**–**4** and the corresponding parent molecules BDT and BDT_1_ were characterized by cyclic voltammetry (CV) in dimethylformamide (DMF) and dichloromethane (DCM) as solvents and 0.1 m tetrabutylammonium perchlorate (TBAP) as supporting electrolyte in a three electrode minicell on a glassy carbon (GC) support as working electrode at potential scan rates of 0.2 V s^−1^, with ohmic drop compensation.The counter electrode was a platinum wire, and a saturated calomel electrode was used as reference electrode inserted in a jacket filled with the same medium used during measurements (DMF or DCM+0.1 m TBAP) to prevent water and chloride leakage.

The experiments were carried out with an AUTOLAB PGSTAT 12(8) potentiostat of EcoChemie (Utrecht, The Netherlands) run by a PC with the GPES 4.9 software of the same manufacturer.

The peak potential values have been normalized versus the [FcH]^+^|FcH intersolvental redox couple currently recommended by IUPAC,^[25]^ having a redox potential of 0.49 V (in DCM) and of 0.51 V (in DMF) versus the operating SCE reference electrode.

**Computational Methods**: Geometries were optimized with B3LYP^[27]^ 6‐31g** level of theory with Grimme's D3 dispersion model..^[21]^ The CPCM[^22^, ^23^] polarizable conductor calculation model was used as a solvation method and dimethylformamide (DMF) was used as solvent. All ground state calculations were carried out using Gaussian program package.^[24]^ Ionization potentials were calculated as vertical and adiabatic detachment energies (VDE and ADE) with the same level and additional diffuse functions were used for the calculation of electron affinities (VEA and AEA). Vertical ionization potentials/electron affinities were calculated as the difference between the energy of neutral molecule and the energy of cation/anion calculated in the same geometry of neutral ones. Adiabatic ionization potentials/electron affinities were calculated as the difference between the energy of neutral molecule and the energy of cation/anion calculated in their optimized geometries.

$$\mathrm{IP}=E{}_{\left(\mathrm{cation}\right)}-E{}_{\left(\mathrm{neutral}\right)}$$

$$\mathrm{EA}=E{}_{\left(\mathrm{anion}\right)}-E{}_{\left(\mathrm{neutral}\right)}$$

Singlet excitations were calculated with CAM‐B3LYP/6‐31g* level^[27]^ using the Q‐Chem 5.1 program package.^[28]^

### Synthesis

#### Benzo[1,2‐b:4,5‐b′]dithiophen‐2‐ylbis(2,4,6 trimethylphenyl)borane (1)

*n*‐BuLi (1.4 m in *n‐*hexane, 0.9 mL, 1.26 mmol, 1.2 equiv.) was added to a solution of BDT_1_ (200 mg, 1.05 mmol) in anhydrous THF (7 mL) cooled to −78 °C. After 1 h, a solution of dimesitylboron fluoride (295 mg, 1.1 mmol, 1.1 equiv.) in THF (2.5 mL) was added. The resulting green suspension was allowed to warm up to room temperature and stirred overnight (the color changed to yellow). The solvent was removed under reduced pressure, brine (15 mL) was added, and the aqueous layer was extracted with CH_2_Cl_2_ (3**×**15 mL). The collected organic phases were washed with H_2_O (15 mL), dried over Na_2_SO_4_ and the solvent was evaporated at reduced pressure affording 470 mg of crude yellow solid that was purified by silica‐gel column chromatography (eluent: *n*‐hexane/CH_2_Cl_2_, 9 : 1). Compound **1** was obtained in 56 % yield as pale yellow solid besides BDT_1_ (10 %) and di‐substituted compound **2** (21 %).

Compound **1** was further purified by washing with pentane.

^1^H NMR (400 MHz, CDCl_3_): *δ*=8.33 (s, 1H), 8.29 (s, 1H), 7.71 (s, 1H), 7.52 (d, *J*=5.5 Hz, 1H), 7.35 (d, *J*=5.6 Hz, 1H), 6.87 (s, 4H), 2.34 (s, 6H), 2.15 ppm (s, 12H). ^13^C{^1^H} NMR (75 MHz, CDCl_3_): *δ*=151.8 (C_q_), 143.5 (C_q_), 141.3 (C_q_), 141.0 (C_q_), 139.1 (C_q_), 138.8 (C_q_), 137.1 (C_q_), 136.1 (CH), 128.7 (CH), 128.3 (CH), 123.0 (CH), 118.9 (CH), 116.9 (CH), 23.3 (CH_3_), 21.3 ppm (CH_3_). ^11^B NMR (128 MHz, CDCl_3_): *δ*=66.0 ppm. EI‐MS m/z: 438 (M+). HRMS (EI) calculated for C_28_H_27_BS_2_: 438.1647, found: 438.1647. M.p.: 246–248 °C.

#### Benzo[1,2‐b:4,5‐b′]dithiophene‐2,6‐diylbis[bis(2,4,6‐trimethylphenyl)borane (2)

*n*‐BuLi (1.2 m in *n*‐hexane, 2.6 ml, 3.15 mmol, 3 equiv.) was added over 5 min to a solution of BDT_1_ (200 mg, 1.05 mmol) in anhydrous THF (7 mL) cooled to −78 °C. The resulting white suspension was allowed to warm up to −10 °C and after 15 min a solution of dimesitylboron fluoride (564 mg, 2.10 mmol, 2 equiv.) in THF (5 mL) was added. The resulting green mixture was thus allowed to warm up to room temperature and stirred overnight. The solvent was removed under reduced pressure, brine (20 mL) was added, and the aqueous layer was extracted with CH_2_Cl_2_ (3**×**15 mL). The collected organic phases were washed with H_2_O (20 mL), dried over Na_2_SO_4_ and the solvent was evaporated affording 730 mg of crude yellow solid that was purified by silica‐gel column chromatography (eluent *n*‐hexane/CH_2_Cl_2_ 9 : 1). Compound **2** was obtained in 78 % yield as bright‐yellow solid, 10 % of **1** was also recovered.

Compound **2** was further purified by washing with diisopropyl ether.

^1^H NMR (300 MHz, CDCl_3_): *δ*=8.31 (s, 2H), 7.68 (s, 2H), 6.85 (s, 8H), 2.33 (s, 12H), 2.13 ppm (s, 24H). ^13^C{^1^H} NMR (75 MHz, CDCl3): *δ*=153.4 (C_q_), 143.2 (Cq), 141.2 (C_q_), 141.0 (C_q_), 140.2 (CH), 139.2 (CH), 135.8 (CH), 128.3 (CH), 118.9 (CH), 23.3 (CH_3_), 21.3 ppm (CH_3_). ^11^B NMR (128 MHz, CDCl_3_): *δ*=73.9 ppm. EI‐MS m/z: 686 (M+). HRMS (EI) calculated for C_46_H_48_B_2_S_2_: 686.3383, found: 686.3382. M.p.: 283–286 °C.

#### Benzo[1,2‐b:4,3‐b′]dithiophen‐2‐ylbis(2,4,6‐trimethylphenyl)borane (3)

Prepared following the same procedure for **1** and obtained in 49 % yield as pale yellow solid. BDT (23 %) and bis‐substituted **4** (19 %) were also recovered.

Compound **3** was further purified by washing with methanol.

^1^H NMR (400 MHz, CDCl_3_): *δ*=8.06 (s, 1H), 7.86 (d, *J*=8.7 Hz, 1H), 7.81 (d, *J*=8.7 Hz, 1H), 7.72 (d, *J*=5.3 Hz, 1H), 7.57 (d, *J*=5.4 Hz, 1H), 6.88 (s, 4H), 2.35 (s, 6H), 2.15 ppm (s, 12H). ^13^C{^1^H} NMR (75 MHz, CDCl_3_): *δ*=150.9 (C_q_), 144.6 (C_q_), 141.3 (C_q_), 141.0 (CH), 139.0 (C_q_), 136.3 (C_q_), 136.1 (C_q_), 135.8 (C_q_), 134.2 (C_q_), 128.3 (CH), 127.0 (CH), 122.3 (CH), 120.7 (CH), 119.0 (CH), 23.4 (CH_3_), 21.3 ppm (CH_3_). ^11^B NMR (128 MHz, CDCl_3_): δ=69.8 ppm. EIMS m/z: 438 (M+). HRMS (EI) calculated for C_28_H_27_BS_2_: 438.1647, found: 438.1649. M.p.: 177–180 °C.

#### Benzo[1,2‐b:4,3‐b′]dithiophene‐2,6‐diylbis[bis(2,4,6‐trimethylphenyl)borane (4)

Prepared following the same procedure for **2** and obtained in 63 % yield as yellow solid. BDT (10 %) and compound **3** (22 %) were also recovered.

Compound **4** was further purified by washing with diisopropyl ether.

^1^H NMR (400 MHz, CDCl_3_): *δ*=8.03 (s, 2H), 7.83 (s, 2H), 6.87 (s, 8H), 2.34 (s, 12H), 2.13 ppm (s, 24H). ^13^C{^1^H} NMR (75 MHz, CDCl_3_): *δ*=151.4 (C_q_), 144.7 (C_q_), 141.3 (C_q_), 141.0 (C_q_), 139.0 (CH), 137.2 (CH), 134.3 (CH), 128.3 (CH), 120.9 (CH), 23.5 (CH_3_), 21.3 ppm (CH_3_). ^11^B NMR (128 MHz, CDCl_3_) *δ* 70.8 ppm. EIMS m/z: 686 (M+). HRMS (EI) calculated for C_46_H_48_B_2_S_2_: 686.3384, found: 686.3385. M.p. 290 °C

## Conflict of interest

The authors declare no conflict of interest.

1

## Supporting information

As a service to our authors and readers, this journal provides supporting information supplied by the authors. Such materials are peer reviewed and may be re‐organized for online delivery, but are not copy‐edited or typeset. Technical support issues arising from supporting information (other than missing files) should be addressed to the authors.

Supporting Information

## Data Availability

The data that support the findings of this study are available from the corresponding author upon reasonable request.
