# Supplementary material for: (Dimesityl)boron Benzodithiophenes: Synthesis, Electrochemical, Photophysical and Theoretical Characterization
Source: ChemistryOpen. 2022 Jan 21;11(1):e202100265. doi: 10.1002/open.202100265 (PMC8780079; doi:10.1002/open.202100265)
Supplement: Supplementary file 1 — Supporting Information [file OPEN-11-e202100265-s001.pdf]

ChemistryOpen

Supporting Information

(Dimesityl)boron Benzodithiophenes: Synthesis, Electrochemical, Photophysical and Theoretical Characterization

Luigi Menduti, Clara Baldoli, Serena Arnaboldi, Andreas Dreuw, Duygu Tahaoglu, Alberto Bossi, and Emanuela Licandro*

Plots of ^{1}H, $^{11}B\{1H\}$ and $^{13}C\{1H\}$ NMR spectra of all new compounds

Compound 1

benzo[1,2-b:4,5-b']dithiophen-2-yldimesitylborane

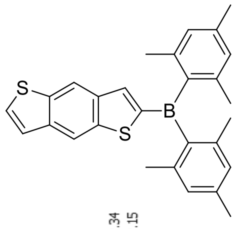

^{1}H NMR

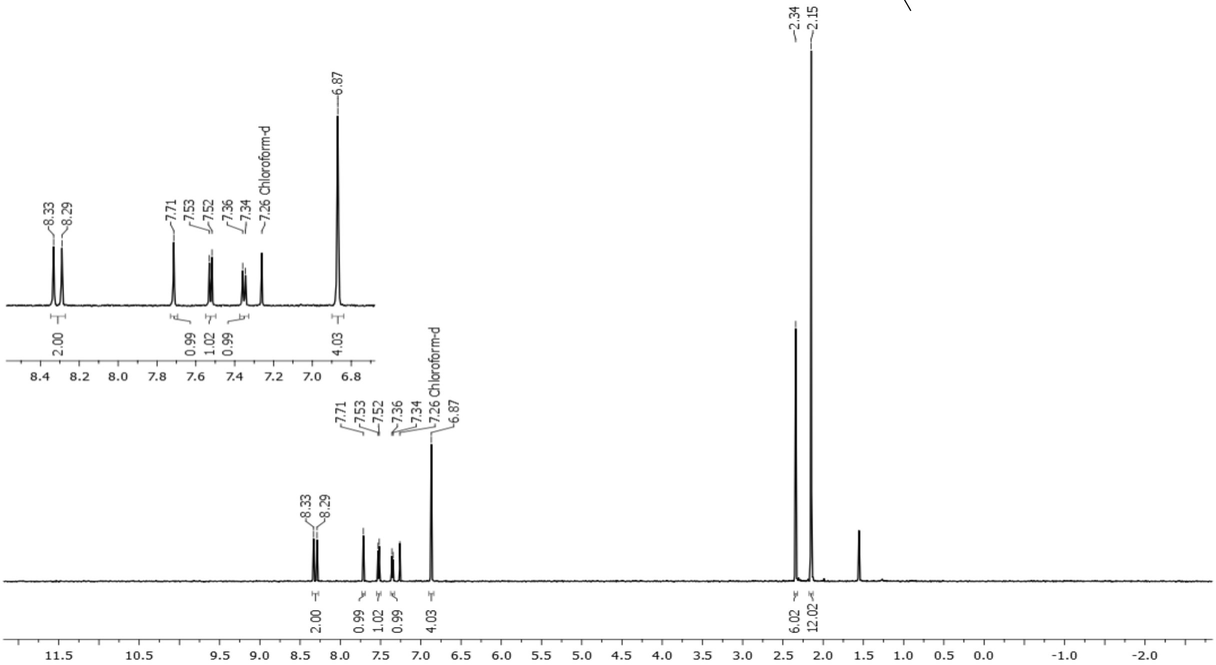

^{13}C NMR

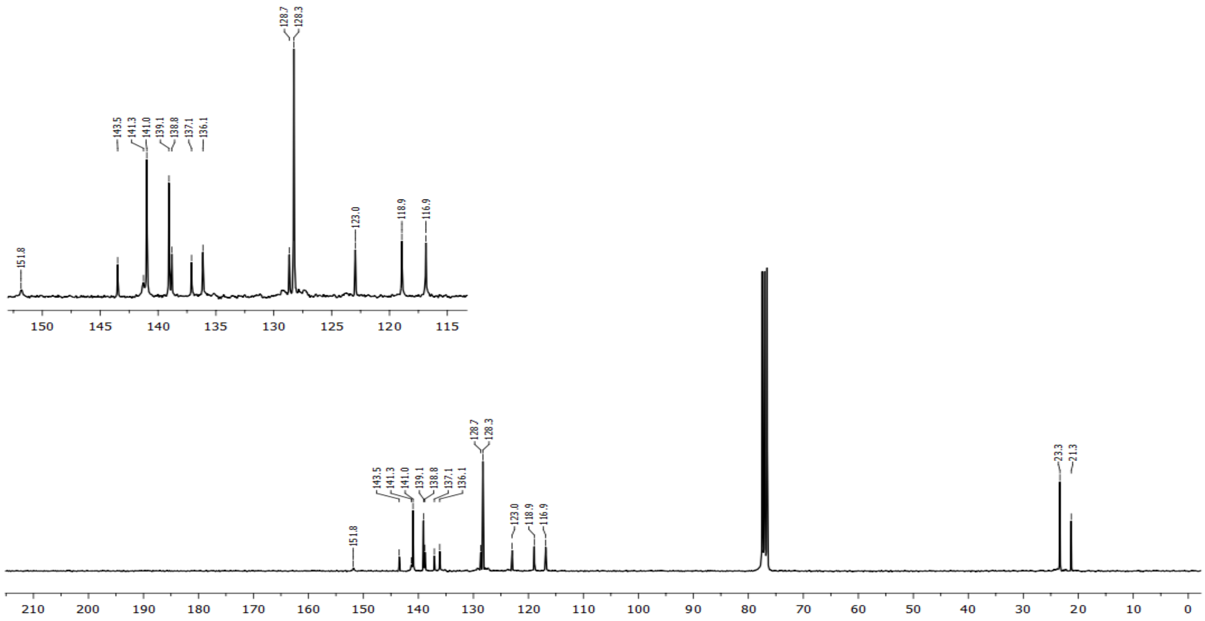

^{11}B NMR

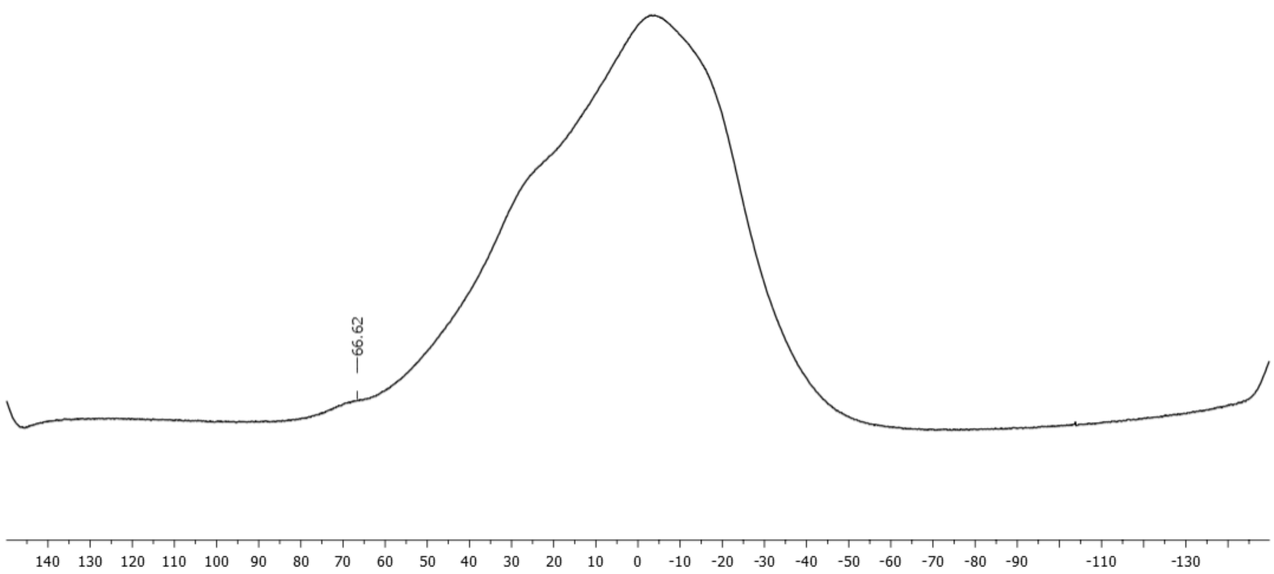

Compound 2

2,6-bis(dimesitylboryl)benzo[1,2-b:4,5-b']dithiophene

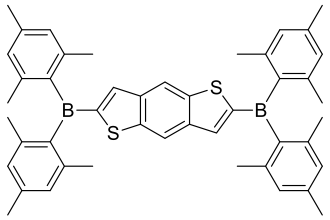

^{1}H NMR

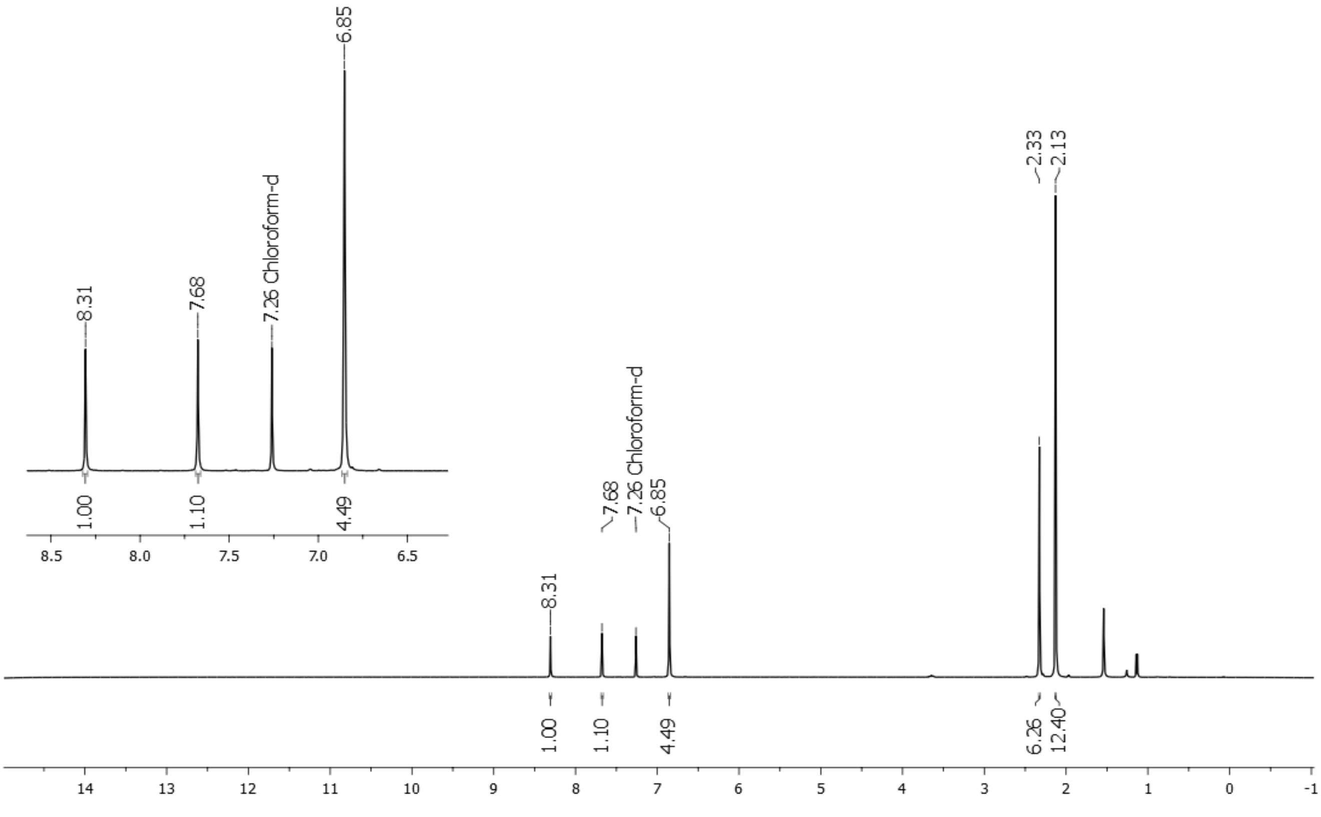

^{13}C NMR

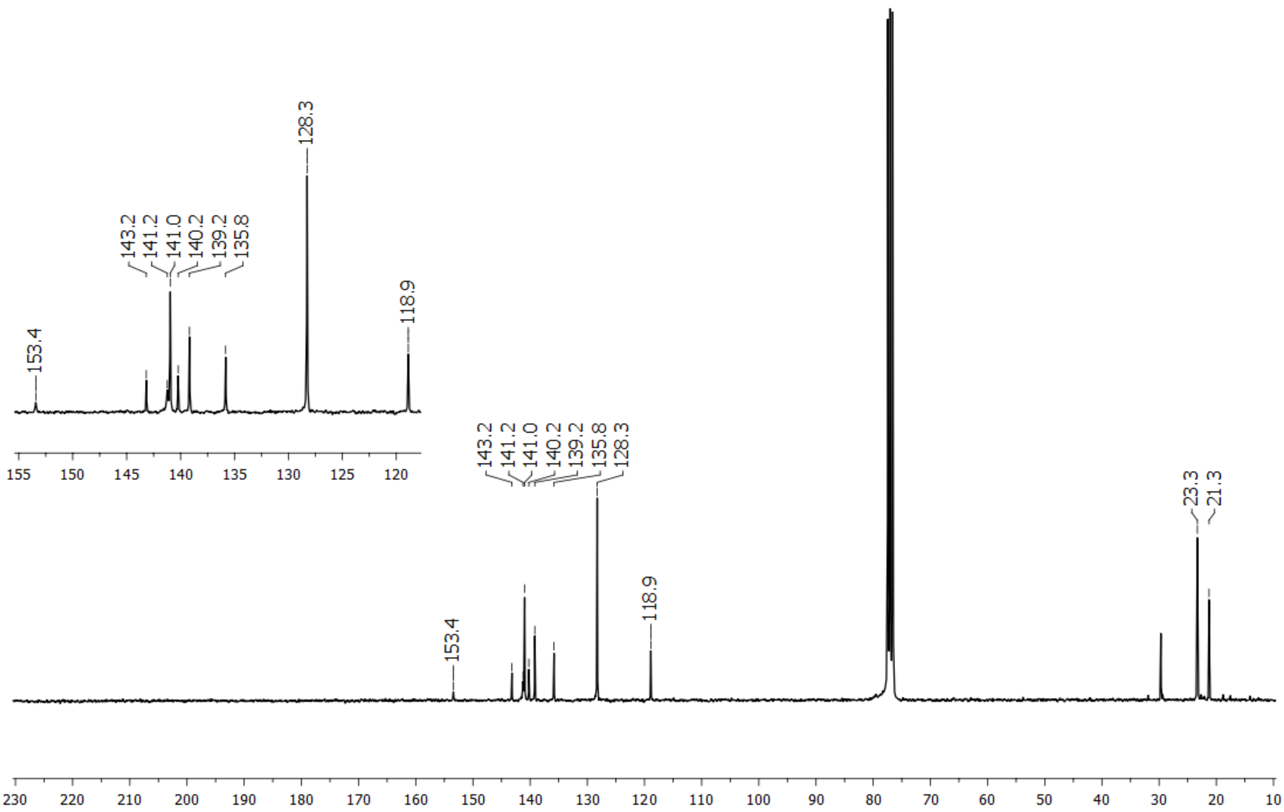

^{11}B NMR

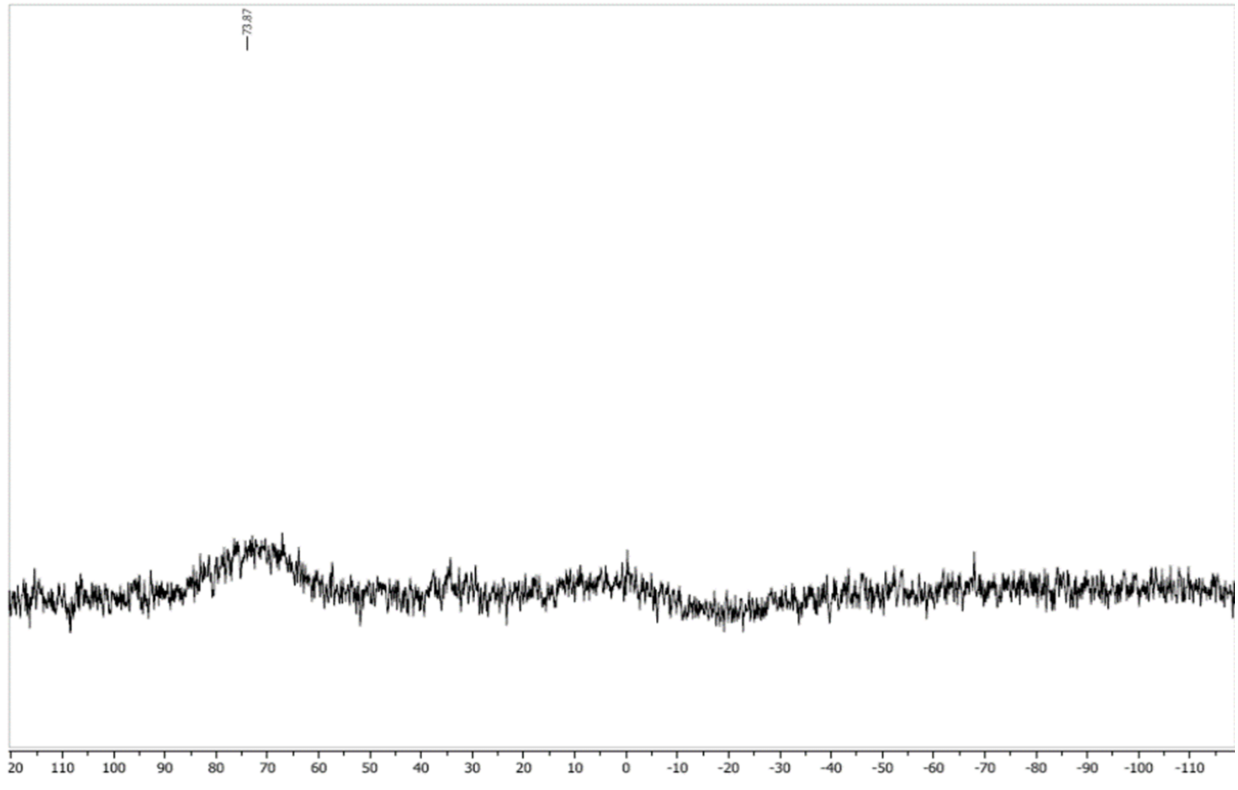

Compound 3 - benzo[1,2-b:4,3-b']dithiophen-2-yldimesitylborane

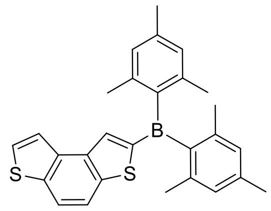

^{1}H NMR

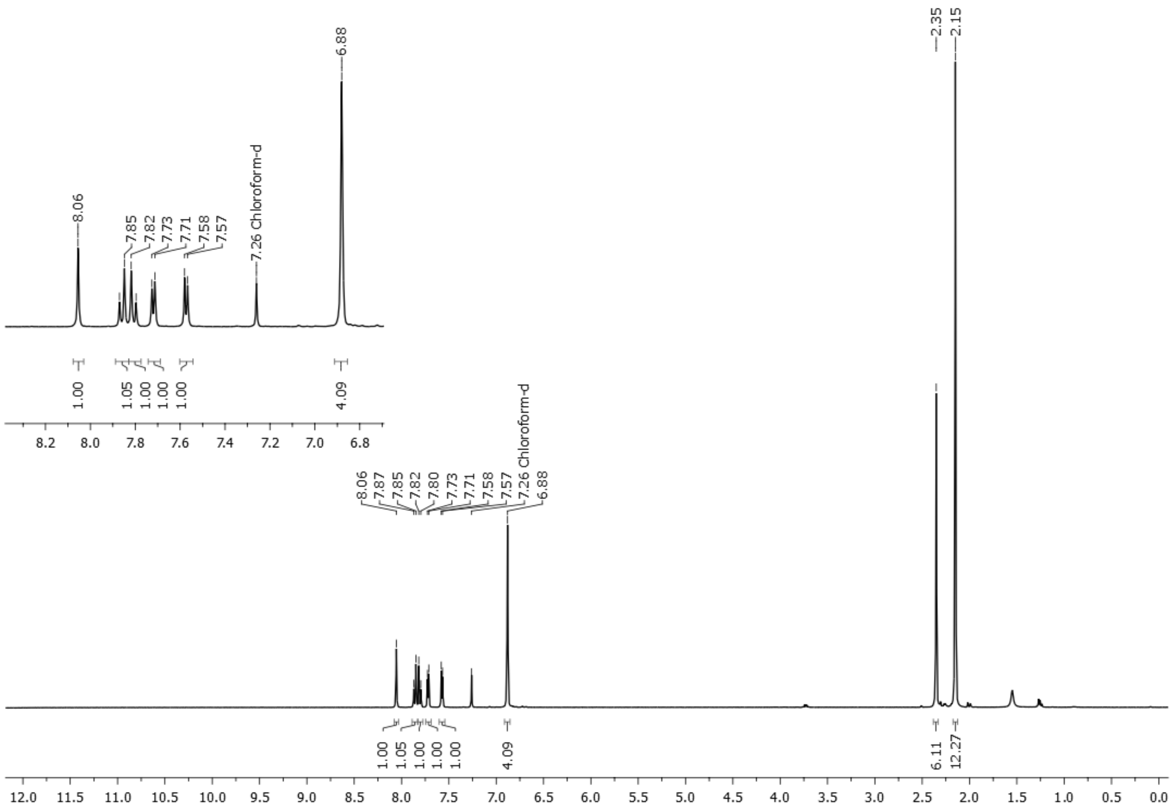

^{13}C NMR

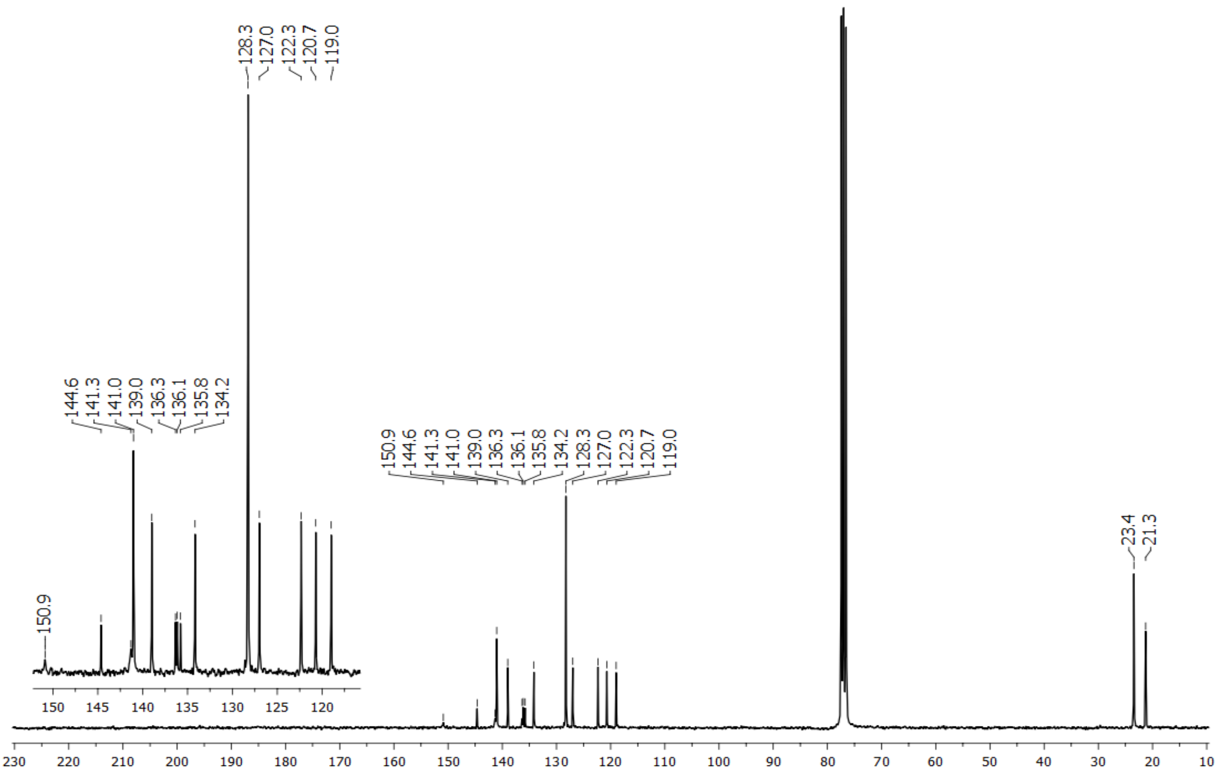

^{11}B NMR

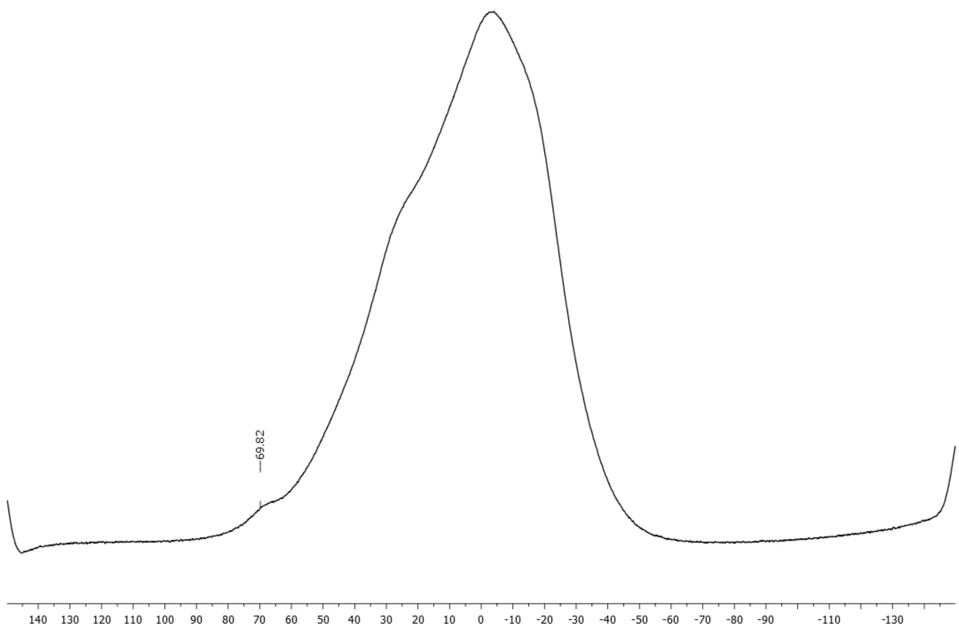

Compound 4 - 2,7-bis(dimesitylboryl)benzo[1,2-b:4,3-b']dithiophene

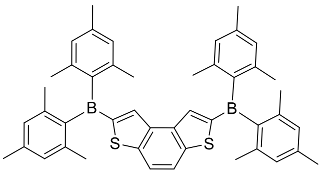

^{1}H NMR

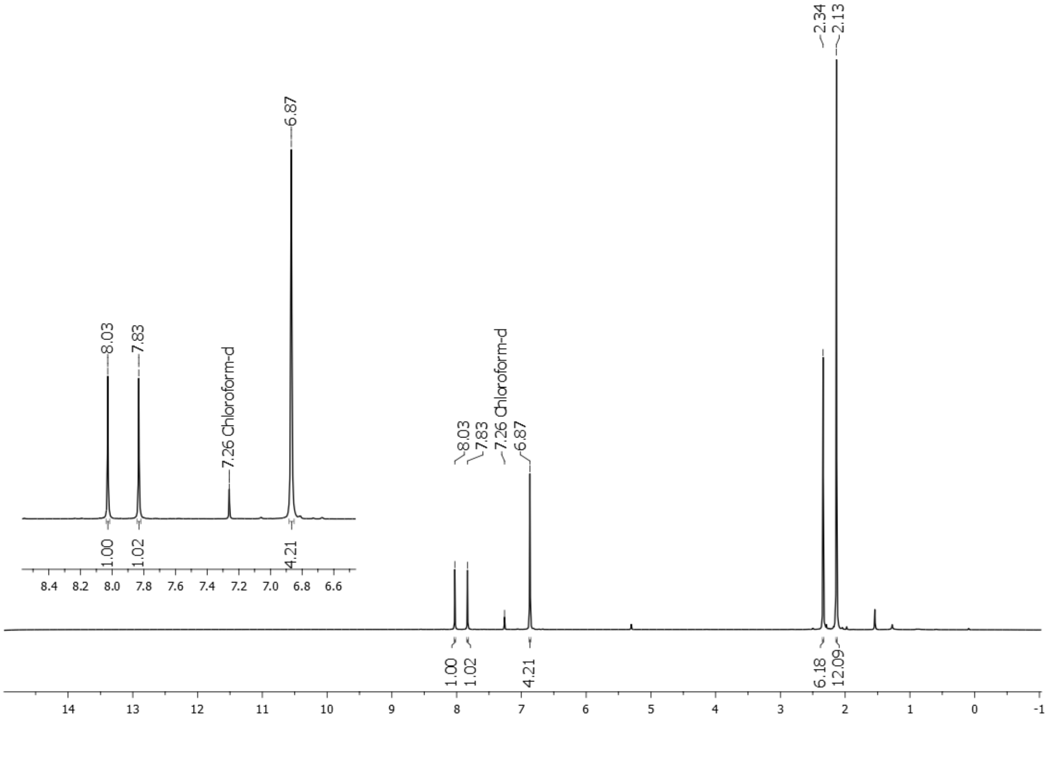

^{13}C NMR

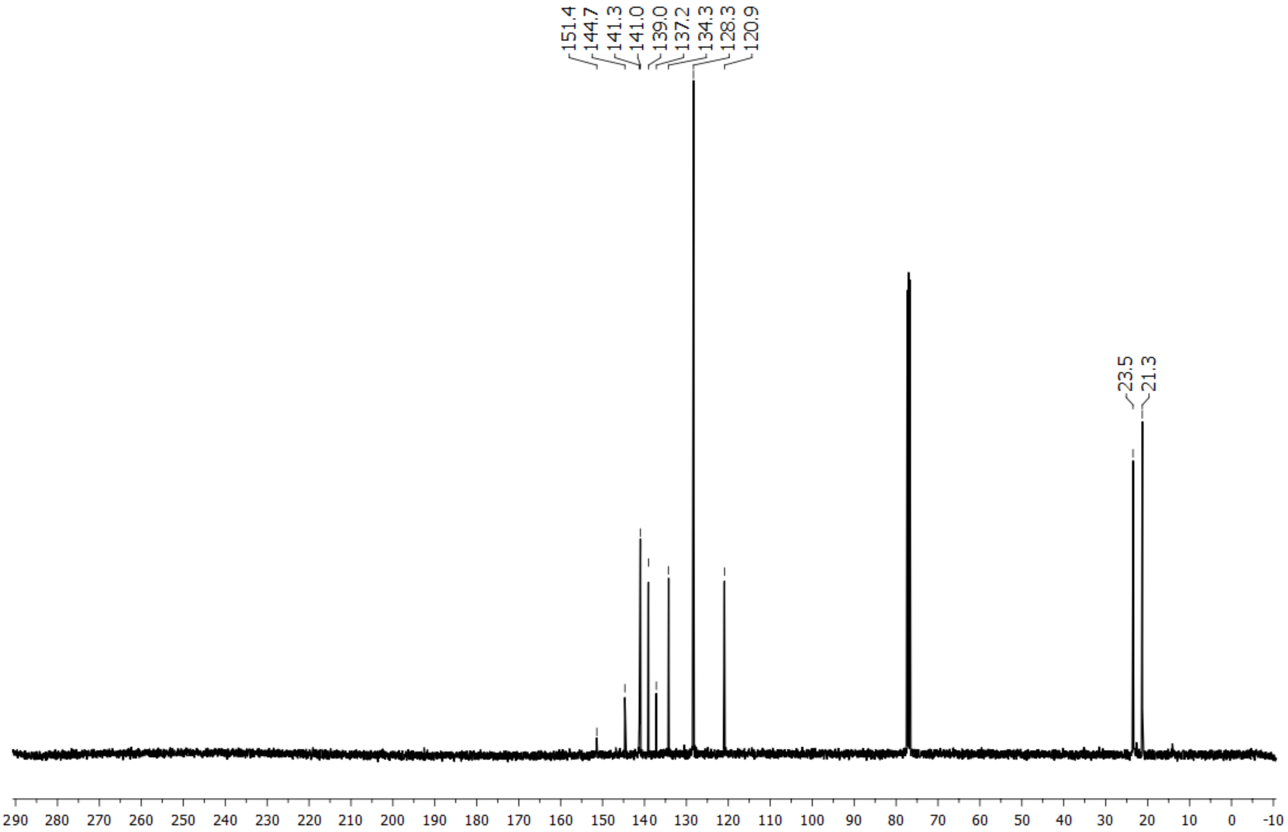

^{11}B NMR

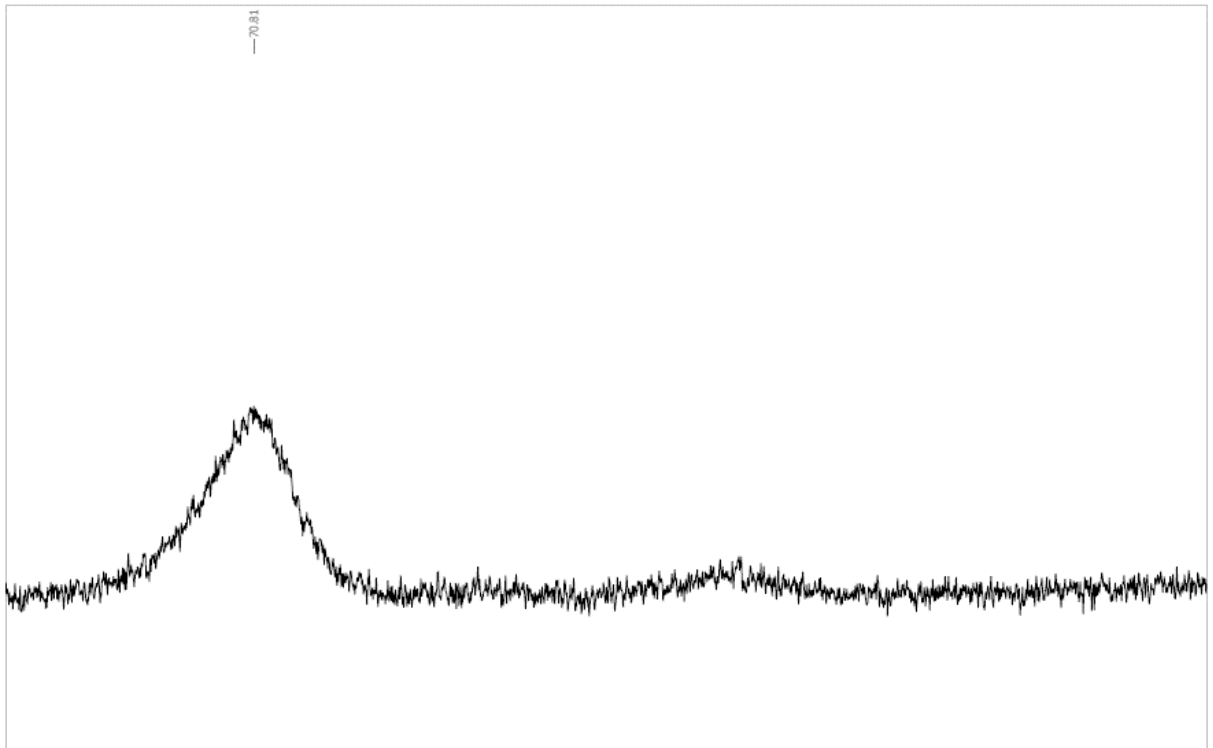

Spectroscopic Section

UV/VIS normalized spectra of monoborylated (**1**,**3**) and diborylated (**2**,**4**) benzodithiophenes in solvent with increasing polarity (hexane <DCM <THF <ACN).

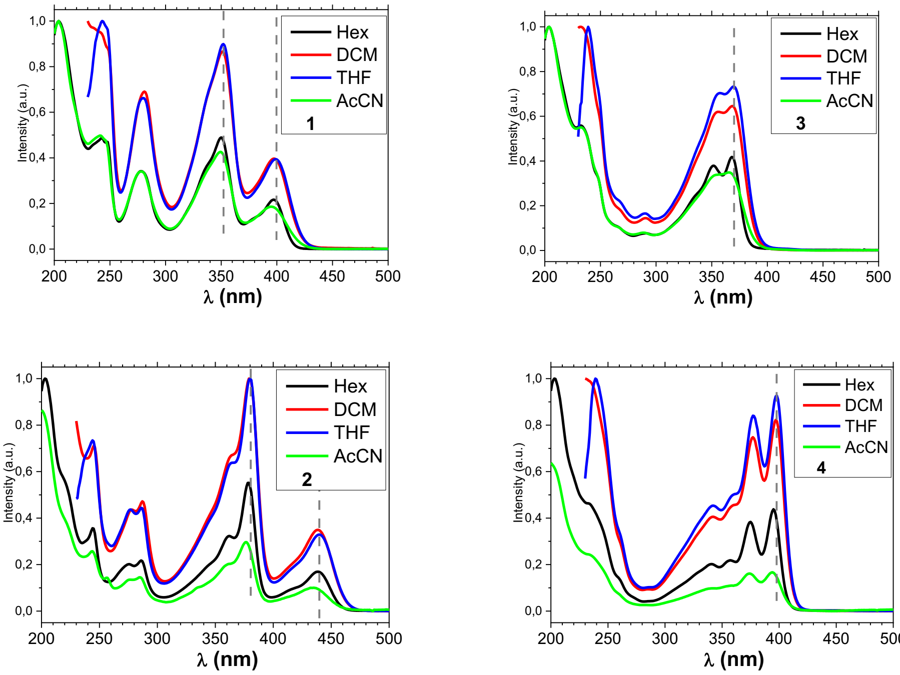

Figure S1: solvatochromic study in Hex, DCM, THF and ACN of the boranes **1-4**.

Vertical dashed lines have been added to better evaluate plots. By varying solvent polarity, no significant solvatochromic effect is observed; this behaviour is compatible with molecular structures without a marked polar (dipolar) character in the ground state. Only a small ipsochromic effect can be detected by comparing absorption spectra in ACN and hexane for compounds **1-4**. This last result lines up with a ground state slightly more polar than the excited one.

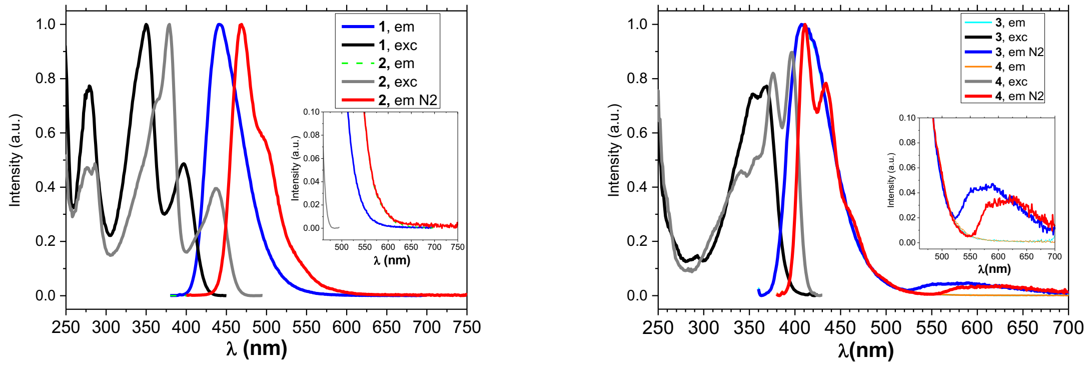

Figure S2: room temperature emission and excitation spectra of boranes ***1-4*** *in DCM solution at r.t.in selected example is reported the PL emission in* N_2 *saturated solutions where a phosphorescent signal is observable.*

Electrochemical Section

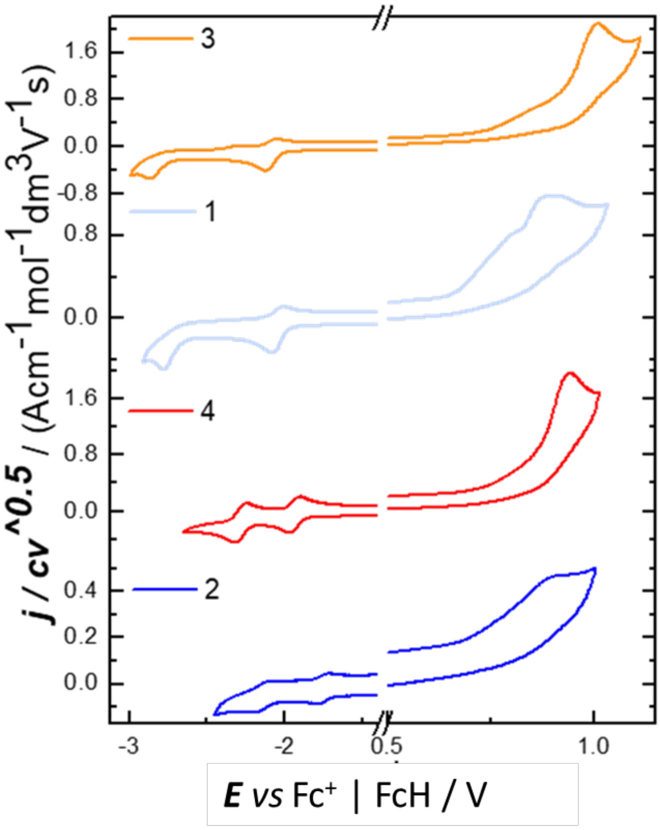

Figure S3: *CV features of compounds* ***1-4*** *recorded in DMF + 0.1 M TBAP at 0.2 V s*$^{-1}$ *potential scan rate on GC electrode.*

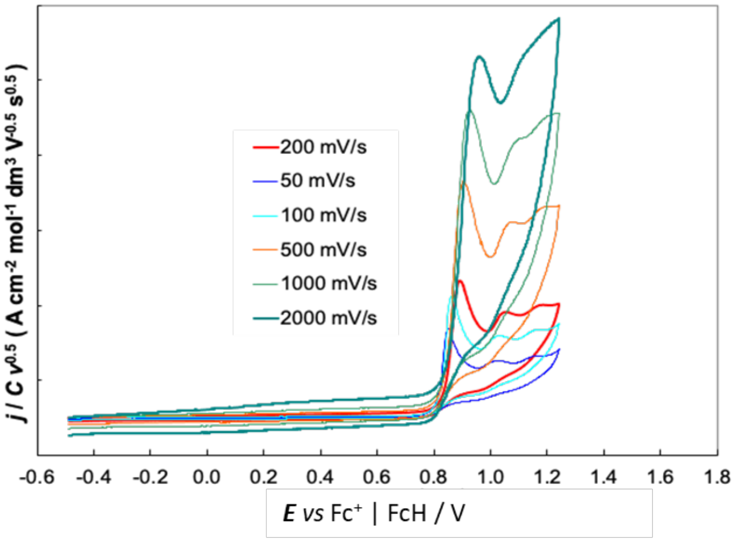

Figure S4: *CV features of the anodic side of compound* **2** *recorded in DCM + 0.1 M TBAP at different potential scan rates (from50 to 2000 mV/s) on GC electrode.*

Spectroscopical, electrochemical and DFT data

	BDT_1	BDT	1	2	3	4
**λ_{abs}[a] [nm] (experim.)**	335	318	398	438	370	398
**λ_{abs}[a][nm] (TD-DFT)**	284	267	343	377	324	352
**HOMO (TD-DFT)**	−5.58	−5.76	−5.62	−5.65	−5.82	−5.87
**LUMO (TD-DFT)**	−1.17	−1.10	−2.13	−2.47	−2.07	−2.36
**HOMO (electrochem)**	−5.81	−5.90	−5.83	−5.70	−5.93	−5.86
**LUMO (electrochem)**	−1.87	−1.80	−2.41	−2.84	−2.38	−2.84
**E_g (TD-DFT)**	4.01	4.66	3.49	3.18	3.75	3.51
**E_g (electrochem)**	3.94	4.10	3.42	2.86	3.55	3.18
**$E_{g\,max}$ (onset)**	3.69	3.90	3.12 (2.88)	2.83 (2.65)	3.37 (3.15)	3.12 (2.98)

Table S1: comparison of spectroscopical, electrochemical and DFT data of compounds **1–4**, **BDT** and **BDT_1**.

**Molecule**	*Attachment Density*	*Detachment Density*
**BDT_1**	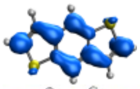	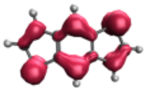
**BDT**	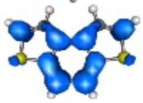	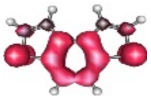
**1**	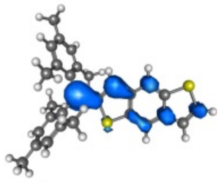	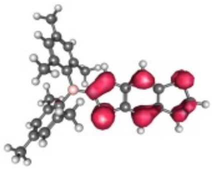
**2**	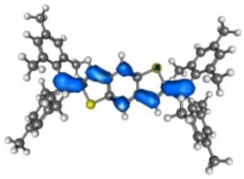	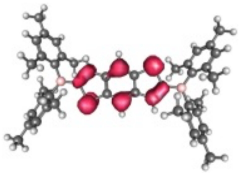
**3**	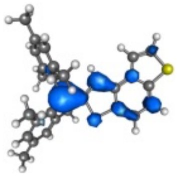	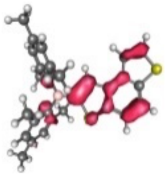
**4**	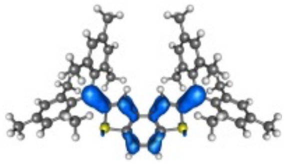	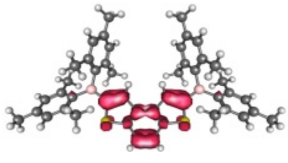

Figure S5: attachment/Detachment densities of the first excited state calculated with CAM-B3LYP/6-31g*.

Cartesian Coordinates of the optimized Structures - DFT/B3LYP-D3/6-311G**/CPCM(DMF)

BDT

S	-3.5935284512	0.3961387932	2.5889376645
C	-3.7439261118	1.6210268225	1.3461795436
C	-2.6408358081	2.4101068039	1.2432369101
C	-1.6073316089	2.0512409939	2.1778090629
C	-1.9775143242	0.9600994954	2.9942112827
C	-0.3250726844	2.6260611644	2.3665828157
C	0.5095092765	2.0794043616	3.3649089474
C	0.1212411176	0.9925507667	4.1720733395
C	-1.1308999754	0.4277819775	3.9844580010
S	2.0426062257	2.9364091523	3.4242295850
C	1.5407552906	3.9973298420	2.1228931558
C	0.2888401970	3.7221375489	1.6698117694
H	-2.5550037914	3.2179265712	0.5261100877
H	-1.4529866333	-0.4128035618	4.5906244861
H	-0.1938866897	4.2682948953	0.8672820682
H	0.7911685535	0.5988289525	4.9291200132
H	2.2187473361	4.7655965148	1.7754786541
H	-4.6548719187	1.6718289058	0.7637626128

BDT Cation

S	-3.6078968526	0.3894087699	2.5867801402
C	-3.7492339074	1.5924756638	1.3703461914
C	-2.6322323353	2.4082720965	1.2509963481
C	-1.6177046985	2.0607848720	2.1632979048
C	-1.9863634879	0.9593460617	2.9899085107
C	-0.3222877260	2.6414914389	2.3539530453
C	0.5159397924	2.0867862755	3.3632221649
C	0.1315360155	1.0183326704	4.1540226533
C	-1.1518345901	0.4385366131	3.9613977295
S	2.0562330803	2.9431226473	3.4269021610
C	1.5591492849	3.9796945088	2.1501483863
C	0.2836641696	3.7133145969	1.6741541013
H	-2.5675598842	3.2110881489	0.5272076551
H	-1.4588761750	-0.4018650769	4.5746266540
H	-0.1792507376	4.2718547768	0.8695882416
H	0.7898757607	0.6136467067	4.9146674924
H	2.2304174653	4.7516071698	1.7950512114
H	-4.6565651743	1.6520620600	0.7814394085

BDT Anion

S	-3.6167100426	0.3794715462	2.5882195402
C	-3.7870898751	1.6182190743	1.3280490209
C	-2.6500996866	2.3992465279	1.2514394404
C	-1.6248637294	2.0517121729	2.1665566813
C	-1.9794624299	0.9464273976	3.0026216546
C	-0.3126656621	2.6393857744	2.3588206431
C	0.5165698194	2.0728221526	3.3763425195
C	0.1410835735	1.0000740943	4.1782615381
C	-1.1492848714	0.4158124773	3.9834449153
S	2.0668870757	2.9450008834	3.4343133129
C	1.5754194084	4.0272051555	2.1145157172
C	0.3025447964	3.7174586338	1.6776699321
H	-2.5578435266	3.2116706028	0.5363068116
H	-1.4675736092	-0.4260401607	4.5910316234
H	-0.1845811159	4.2604136704	0.8720496995

H	0.8091093221	0.6055635317	4.9376387139
H	2.2503712614	4.7955978024	1.7665424271
H	-4.6948007080	1.6699186632	0.7438858085

BDT1

S	-3.8014418917	1.0212973569	-0.0000313518
C	-4.5682804613	2.6029865710	-0.0000367639
C	-3.6809466073	3.6296402883	-0.0000385587
C	-2.3060276346	3.1907494282	-0.0000339197
C	-2.2019819547	1.7662989251	-0.0000293609
C	-1.1408055819	3.9684957196	-0.0000336850
C	0.0823323249	3.3076263921	-0.0000287232
C	0.1860254196	1.8837661456	-0.0000239310
C	-0.9795269080	1.1052765330	-0.0000244879
S	1.6821123272	4.0480082255	-0.0000279824
C	2.4475005366	2.4655839425	-0.0000196727
C	1.5580991859	1.4415747140	-0.0000194298
H	-5.6494109447	2.6585712732	-0.0000391025
H	-3.9752459175	4.6729961028	-0.0000425611
H	-1.2052326133	5.0517002996	-0.0000374776
H	-0.9204387265	0.0209138229	-0.0000212848
H	3.5279217093	2.4056332663	-0.0000160821
H	1.8447277380	0.3958709932	-0.0000156249

BDT1 Cation

S	-3.8042116303	1.0306232703	-0.0000394928
C	-4.5521312673	2.5786450384	-0.0000318418
C	-3.6519928871	3.6292168646	-0.0000384449
C	-2.3060723224	3.1867041396	-0.0000362420
C	-2.2067227378	1.7636369720	-0.0000321554
C	-1.1259888587	3.9750714568	-0.0000354977
C	0.0871351467	3.3103373653	-0.0000316440
C	0.1860793352	1.8878171382	-0.0000271539
C	-0.9943474087	1.0984985216	-0.0000270315
S	1.6848929956	4.0388913668	-0.0000384239
C	2.4314158843	2.4899781459	-0.0000142994
C	1.5294072242	1.4422461745	-0.0000199929
H	-5.6330084665	2.6459771331	-0.0000285662
H	-3.9540100917	4.6694138684	-0.0000391034
H	-1.1945101325	5.0574678008	-0.0000395802
H	-0.9317285740	0.0148203017	-0.0000240035
H	3.5114887300	2.4180215398	-0.0000040920
H	1.8236850611	0.3996229020	-0.0000124346

BDT1 Anion

S	-3.8209620860	1.0127796774	-0.0000307237
C	-4.6203614139	2.6013282036	-0.0000374859
C	-3.6876077098	3.6207886932	-0.0000385573
C	-2.3344140134	3.1953091701	-0.0000338917
C	-2.2053569024	1.7639285592	-0.0000291349
C	-1.1271490518	3.9758897146	-0.0000333615
C	0.0850797869	3.3092855592	-0.0000284503
C	0.2138375075	1.8784703155	-0.0000237223
C	-0.9936201117	1.0970446390	-0.0000241696
S	1.7008192695	4.0567306231	-0.0000271275
C	2.4993005896	2.4678112799	-0.0000201827
C	1.5651348739	1.4507215068	-0.0000193900

H	-5.6995232335	2.6573987307	-0.0000403212
H	-3.9757193360	4.6686478931	-0.0000427868
H	-1.1820366052	5.0605691293	-0.0000368762
H	-0.9432159436	0.0113566052	-0.0000208066
H	3.5778849330	2.4078882765	-0.0000173509
H	1.8472894470	0.4010414235	-0.0000156607

1

S	-3.8513305458	1.0315032916	0.0170516964
C	-4.7292545182	2.5745973725	0.1171373326
C	-3.8650981215	3.6080060342	0.3997256355
C	-2.4961316720	3.2184787314	0.5662703085
C	-2.3217766499	1.8113091717	0.3748203348
C	-1.3892630237	4.0202101670	0.8924234271
C	-0.1547960100	3.3985156105	1.0127791073
C	0.0208752724	1.9929215508	0.8135264960
C	-1.0811856096	1.1936877382	0.4871308947
S	1.3797562352	4.1623907357	1.4282856949
C	2.2091653790	2.6166924763	1.3301851296
C	1.3891347474	1.5864548554	1.0041744439
B	-6.2691757222	2.6233419650	-0.0703077967
H	-4.2101878721	4.6314148113	0.4983117409
H	-1.5120391804	5.0866418505	1.0496620184
H	-0.9658464820	0.1240632488	0.3405491292
H	3.2726105476	2.5786727362	1.5271325730
H	1.7225897132	0.5600630877	0.9002648892
C	-6.9413921374	4.0310455919	-0.3025291047
C	-7.9264895121	4.5135689581	0.6047589524
C	-8.3833174044	3.6823700345	1.7845769064
C	-8.4774691229	5.7828107221	0.4291523424
H	-9.2151225127	6.1418454023	1.1442696144
C	-8.1150247954	6.6046797883	-0.6472979257
C	-8.7534946691	7.9604708817	-0.8287020899
C	-7.1706525753	6.1177381447	-1.5478768380
H	-6.8848092632	6.7287706751	-2.4021844275
C	-6.5805862136	4.8541878437	-1.3943853606
C	-5.6304481837	4.4061859201	-2.4883922110
C	-7.0955221759	1.2762930821	-0.0297024764
C	-7.1239410221	0.4440635005	1.1185401957
C	-6.4572836891	0.8259866736	2.4245974780
C	-7.8464897949	-0.7549398162	1.1035803166
H	-7.8607699384	-1.3692104650	2.0021803339
C	-8.5475849188	-1.1793624827	-0.0264450910
C	-9.3062681958	-2.4850326758	-0.0267230348
C	-8.5174702129	-0.3575082293	-1.1575932786
H	-9.0530129229	-0.6705841554	-2.0515674564
C	-7.8190348403	0.8531499644	-1.1767457722
C	-7.8254656942	1.6632550420	-2.4564564177
H	-9.1413640318	4.2117116921	2.3680623075
H	-7.5536730309	3.4413585878	2.4569753557
H	-8.8113804420	2.7305573221	1.4557692121
H	-8.2933473827	8.5131875491	-1.6525074104
H	-8.6663340587	8.5645298346	0.0812039827
H	-9.8240852706	7.8628220108	-1.0454263762
H	-4.8384072918	5.1458000384	-2.6466124902
H	-6.1712927268	4.3039636261	-3.4367456570
H	-5.1558540624	3.4483786634	-2.2770983928

H	-5.8688703087	-0.0087817671	2.8196890713
H	-7.2145573360	1.0732995814	3.1777515488
H	-5.7961772577	1.6874459715	2.3283417014
H	-9.8603234868	-2.6251038293	-0.9592236954
H	-10.0204744834	-2.5299820867	0.8031519566
H	-8.6257799869	-3.3366327540	0.0917143296
H	-8.1971673153	2.6781471810	-2.2892557228
H	-8.4553867495	1.1901518649	-3.2146310205
H	-6.8182614701	1.7588966783	-2.8763464106

1 Cation

S	-3.8943939062	1.0911717445	-0.0879477082
C	-4.7404035862	2.5981679249	0.0853304722
C	-3.8625189228	3.6345689148	0.4202462496
C	-2.5188179160	3.2211295445	0.5711898982
C	-2.3598699352	1.8259081572	0.3125925667
C	-1.3946856258	3.9979874955	0.9532039885
C	-0.1752800552	3.3500770085	1.0583998610
C	-0.0165170927	1.9569602678	0.7942989719
C	-1.1374819658	1.1813899627	0.4076959425
S	1.3561220530	4.0621967755	1.5353736659
C	2.1539028054	2.5437829370	1.3818528325
C	1.3182394229	1.5203818144	0.9843714686
B	-6.3029604704	2.6488091622	-0.0891590872
H	-4.2059419151	4.6525920620	0.5623598757
H	-1.5068793947	5.0567692018	1.1601272314
H	-1.0324422758	0.1193034191	0.2097639569
H	3.2128267941	2.4738698666	1.5952130906
H	1.6478151927	0.4985387787	0.8394128526
C	-6.9646096886	4.0446355527	-0.3405687754
C	-7.9837033913	4.5116771788	0.5388948476
C	-8.4989391143	3.6609830395	1.6804439225
C	-8.5073990174	5.7932403768	0.3712457933
H	-9.2646926583	6.1495970771	1.0661370899
C	-8.0922770184	6.6337998233	-0.6710356780
C	-8.7139494161	7.9960968355	-0.8548523136
C	-7.1211546634	6.1567109894	-1.5506766927
H	-6.7992773087	6.7829579211	-2.3803947727
C	-6.5548974817	4.8843089884	-1.4043117705
C	-5.5855054879	4.4314530461	-2.4772460664
C	-7.0974712395	1.2966143648	-0.0056138878
C	-7.0603493225	0.4629233871	1.1401458762
C	-6.3866503271	0.8756935358	2.4320146217
C	-7.7427869737	-0.7608842377	1.1419684507
H	-7.7125556270	-1.3770141512	2.0380881808
C	-8.4640950836	-1.2002023300	0.0331679980
C	-9.1699350177	-2.5338599913	0.0239968407
C	-8.5055709347	-0.3691769822	-1.0952739293
H	-9.0628385326	-0.6984490162	-1.9698055151
C	-7.8539068357	0.8624710357	-1.1324953156
C	-7.9346781580	1.6830218020	-2.4031638390
H	-9.2608891169	4.1961416979	2.2530234949
H	-7.6995049614	3.3790272023	2.3725833872
H	-8.9421647911	2.7310372379	1.3121567782
H	-8.0940813717	8.6376934136	-1.4873971814
H	-8.8658983814	8.4963756787	0.1067247443
H	-9.6978631036	7.9105580185	-1.3328655417

H	-4.7936048988	5.1712819567	-2.6318954486
H	-6.1132239393	4.3155541048	-3.4314060409
H	-5.1137278570	3.4729971632	-2.2564759087
H	-5.7677588189	0.0643387075	2.8286104845
H	-7.1436401703	1.1060575082	3.1905970376
H	-5.7574217956	1.7608473392	2.3263746281
H	-10.1523953771	-2.4571524223	-0.4532576130
H	-9.3048403366	-2.9261220880	1.0356831930
H	-8.5937907416	-3.2737700046	-0.5454878555
H	-8.3590977857	2.6735236258	-2.2171506428
H	-8.5549301882	1.1806817581	-3.1498966182
H	-6.9449762725	1.8392258194	-2.8459220911

1 Anion

S	-3.8175593451	1.0261613529	0.1616024357
C	-4.7105702405	2.6014040816	0.1446825218
C	-3.7703173336	3.6339837048	0.3363724911
C	-2.4223666358	3.2370840451	0.5128822045
C	-2.2608142831	1.8055604029	0.4514140579
C	-1.2747275725	4.0327293854	0.7530874099
C	-0.0498555416	3.3934221144	0.9156460313
C	0.1029816664	1.9774475367	0.8499133977
C	-1.0404703059	1.1789391317	0.6131612733
S	1.5130874913	4.1612719755	1.2328195492
C	2.3230847731	2.5943788351	1.2692067201
C	1.4613223478	1.5627800647	1.0546017859
B	-6.2081474925	2.6324450701	-0.0535023450
H	-4.0867915033	4.6712514569	0.3823241689
H	-1.3633030443	5.1130575928	0.8122272362
H	-0.9518328646	0.0966425654	0.5672447064
H	3.3885033184	2.5515324198	1.4499968325
H	1.7694293553	0.5221038112	1.0415720283
C	-6.9274202976	4.0383232222	-0.2844347594
C	-7.9714369368	4.4825380338	0.5756648742
C	-8.4358916872	3.6347199979	1.7410599332
C	-8.5902529452	5.7206429628	0.3761909399
H	-9.3754666930	6.0359989151	1.0622796225
C	-8.2309398038	6.5653708874	-0.6801858458
C	-8.9398165499	7.8814562297	-0.8998246830
C	-7.2202297527	6.1286382202	-1.5373584108
H	-6.9299653964	6.7598048587	-2.3763220180
C	-6.5734819302	4.8959704885	-1.3611692465
C	-5.5125128222	4.5140780781	-2.3735442131
C	-7.0644538324	1.2792230438	-0.0223336335
C	-7.1414609787	0.4541064062	1.1301988404
C	-6.4309267156	0.7896179481	2.4261164510
C	-7.9179111958	-0.7156728691	1.1308924214
H	-7.9601899436	-1.3156783584	2.0391426935
C	-8.6391748206	-1.1255778527	0.0111095690
C	-9.4443247783	-2.4041854761	0.0057162644
C	-8.5711520285	-0.3155650733	-1.1289107155
H	-9.1244064463	-0.6121711041	-2.0190642747
C	-7.8105151324	0.8562879295	-1.1604883972
C	-7.7726201702	1.6380330172	-2.4567678273
H	-9.1915293878	4.1568534277	2.3361033067
H	-7.6051972705	3.3701920403	2.4026966994
H	-8.8665376942	2.6887811223	1.3978458269

H	-8.3866706818	8.5240412200	-1.5916540163
H	-9.0721592603	8.4268222953	0.0411188337
H	-9.9413482871	7.7282810857	-1.3224077381
H	-4.5026899960	4.6821540567	-1.9847522143
H	-5.6214542375	5.0977311889	-3.2935439504
H	-5.5649726771	3.4525457037	-2.6266655143
H	-5.6588898373	0.0453395180	2.6534192057
H	-7.1360498474	0.7895863861	3.2658424870
H	-5.9452682652	1.7641493446	2.3802871928
H	-10.3998623963	-2.2730193551	-0.5141895758
H	-9.6530998459	-2.7532157189	1.0215317425
H	-8.9075016999	-3.2085328074	-0.5138423607
H	-8.0513730987	2.6854204646	-2.3110975603
H	-8.4461167131	1.2030829325	-3.2018417613
H	-6.7627207355	1.6401320422	-2.8830806934

2

C	1.11773	0.77603	0.27096
C	1.26444	-0.56795	-0.20412
C	2.64201	-0.93914	-0.31451
C	3.53632	0.04315	0.03487
C	-0.13087	1.34823	0.47617
C	0.12993	-1.34581	-0.48629
C	-1.11868	-0.77360	-0.28113
C	-1.26539	0.57021	0.19443
H	-0.23990	2.36581	0.83685
H	0.23896	-2.36347	-0.84675
C	-2.64299	0.94070	0.30676
H	-2.96970	1.91870	0.64227
C	-3.53733	-0.04184	-0.04190
S	-2.68736	-1.51984	-0.54411
S	2.68644	1.52200	0.53467
H	2.96870	-1.91765	-0.64856
B	-5.08572	0.04262	-0.00340
C	-5.92722	-1.29047	-0.04126
C	-6.84295	-1.53366	-1.10261
C	-5.77787	-2.28785	0.95307
C	-7.54568	-2.73790	-1.15705
C	-6.51222	-3.47924	0.87047
C	-7.39488	-3.72930	-0.17861
H	-8.23153	-2.91211	-1.98366
H	-6.39128	-4.22507	1.65382
C	-5.73758	1.47669	0.06227
C	-6.61833	1.81531	1.12519
C	-5.46569	2.46073	-0.92059
C	-7.17308	3.09503	1.19428
C	-6.06257	3.72489	-0.83068
C	-6.90966	4.06827	0.22360
H	-7.83508	3.34039	2.02209
H	-5.86006	4.45852	-1.60852
C	-6.95326	0.82316	2.21945
H	-6.05409	0.48162	2.74290
H	-7.61881	1.26864	2.96355
H	-7.44166	-0.06855	1.81599
C	-7.51063	5.44901	0.32953
H	-8.52869	5.41183	0.72984
H	-6.92169	6.08102	1.00586

H	-7.54103	5.94964	-0.64244
C	-4.56248	2.20484	-2.11347
H	-4.94422	2.71977	-3.00048
H	-3.54967	2.57981	-1.92796
H	-4.46606	1.14582	-2.35637
C	-4.91099	-2.09797	2.18188
H	-4.28211	-2.97671	2.35795
H	-4.25754	-1.22744	2.11393
H	-5.53943	-1.96788	3.07101
C	-8.15512	-5.02987	-0.27694
H	-7.70984	-5.68770	-1.03341
H	-8.15215	-5.57035	0.67363
H	-9.19577	-4.86119	-0.57279
C	-7.04857	-0.52601	-2.21413
H	-7.31917	0.45792	-1.82188
H	-6.13644	-0.39234	-2.80686
H	-7.83720	-0.85070	-2.89802
B	5.08472	-0.04272	0.00078
C	5.92742	1.28960	0.03766
C	6.84002	1.53486	1.10123
C	5.78175	2.28462	-0.95964
C	7.54359	2.73866	1.15465
C	6.51691	3.47556	-0.87793
C	7.39665	3.72758	0.17313
H	8.22697	2.91452	1.98295
H	6.39889	4.21952	-1.66350
C	5.73584	-1.47745	-0.05834
C	5.45959	-2.45837	0.92614
C	6.61511	-1.82175	-1.12082
C	6.05200	-3.72497	0.83912
C	7.16544	-3.10340	-1.18688
C	6.90223	-4.07190	-0.21121
H	5.84149	-4.45841	1.61508
H	7.82124	-3.35528	-2.01769
C	7.04104	0.53022	2.21632
H	7.82764	0.85622	2.90192
H	6.12678	0.39906	2.80634
H	7.31209	-0.45507	1.82784
C	8.15759	5.02782	0.27038
H	7.70961	5.68857	1.02270
H	9.19681	4.85926	0.57128
H	8.15917	5.56512	-0.68199
C	4.91820	2.09226	-2.19039
H	4.29379	2.97283	-2.37301
H	5.54903	1.95489	-3.07674
H	4.26081	1.22489	-2.12024
C	4.55169	-2.19901	2.11465
H	4.45399	-1.13916	2.35340
H	3.53974	-2.57509	1.92663
H	4.93036	-2.71060	3.00489
C	7.54760	-5.43485	-0.28128
H	8.58189	-5.39782	0.08257
H	7.01036	-6.16564	0.32987
H	7.58285	-5.80674	-1.31025
C	6.94714	-0.83530	-2.22112
H	6.04700	-0.49820	-2.74585
H	7.43420	0.05947	-1.82283

H	7.61312	-1.28358	-2.96315

2 Cation

C	1.117731	0.776031	0.270963
C	1.264439	-0.567947	-0.204118
C	2.642015	-0.939140	-0.314507
C	3.536319	0.043146	0.034866
C	-0.130873	1.348226	0.476175
C	0.129929	-1.345811	-0.486289
C	-1.118679	-0.773597	-0.281130
C	-1.265390	0.570206	0.194435
H	-0.239902	2.365805	0.836855
H	0.238962	-2.363467	-0.846750
C	-2.642989	0.940699	0.306760
H	-2.969701	1.918696	0.642275
C	-3.537332	-0.041840	-0.041898
S	-2.687358	-1.519840	-0.544111
S	2.686441	1.522002	0.534674
H	2.968698	-1.917648	-0.648564
B	-5.085720	0.042617	-0.003397
C	-5.927218	-1.290474	-0.041259
C	-6.842945	-1.533663	-1.102614
C	-5.777875	-2.287853	0.953068
C	-7.545679	-2.737903	-1.157048
C	-6.512223	-3.479241	0.870466
C	-7.394879	-3.729297	-0.178606
H	-8.231526	-2.912113	-1.983658
H	-6.391277	-4.225066	1.653824
C	-5.737584	1.476688	0.062270
C	-6.618335	1.815311	1.125194
C	-5.465690	2.460727	-0.920588
C	-7.173078	3.095033	1.194284
C	-6.062573	3.724891	-0.830676
C	-6.909663	4.068267	0.223597
H	-7.835084	3.340388	2.022089
H	-5.860055	4.458522	-1.608523
C	-6.953263	0.823158	2.219448
H	-6.054088	0.481620	2.742904
H	-7.618809	1.268643	2.963550
H	-7.441659	-0.068554	1.815991
C	-7.510628	5.449013	0.329526
H	-8.528694	5.411828	0.729845
H	-6.921685	6.081017	1.005865
H	-7.541031	5.949638	-0.642443
C	-4.562485	2.204841	-2.113472
H	-4.944223	2.719768	-3.000481
H	-3.549667	2.579809	-1.927963
H	-4.466061	1.145821	-2.356374
C	-4.910985	-2.097973	2.181881
H	-4.282112	-2.976708	2.357948
H	-4.257537	-1.227443	2.113927
H	-5.539431	-1.967879	3.071012
C	-8.155118	-5.029872	-0.276935
H	-7.709840	-5.687699	-1.033408
H	-8.152149	-5.570348	0.673632
H	-9.195772	-4.861187	-0.572791
C	-7.048569	-0.526013	-2.214133

H	-7.319174	0.457916	-1.821882
H	-6.136435	-0.392339	-2.806857
H	-7.837196	-0.850696	-2.898019
B	5.084716	-0.042723	0.000783
C	5.927420	1.289604	0.037656
C	6.840019	1.534861	1.101231
C	5.781755	2.284620	-0.959642
C	7.543590	2.738664	1.154649
C	6.516913	3.475564	-0.877933
C	7.396645	3.727580	0.173133
H	8.226971	2.914520	1.982949
H	6.398893	4.219524	-1.663504
C	5.735841	-1.477445	-0.058338
C	5.459594	-2.458371	0.926140
C	6.615112	-1.821752	-1.120822
C	6.052005	-3.724967	0.839118
C	7.165443	-3.103399	-1.186880
C	6.902234	-4.071903	-0.211205
H	5.841487	-4.458412	1.615080
H	7.821237	-3.355285	-2.017693
C	7.041044	0.530221	2.216317
H	7.827638	0.856216	2.901917
H	6.126783	0.399064	2.806337
H	7.312093	-0.455074	1.827845
C	8.157593	5.027822	0.270381
H	7.709607	5.688571	1.022701
H	9.196813	4.859257	0.571278
H	8.159167	5.565117	-0.681994
C	4.918203	2.092264	-2.190395
H	4.293787	2.972828	-2.373005
H	5.549033	1.954890	-3.076741
H	4.260808	1.224888	-2.120243
C	4.551688	-2.199006	2.114645
H	4.453994	-1.139157	2.353402
H	3.539740	-2.575086	1.926628
H	4.930357	-2.710604	3.004891
C	7.547601	-5.434854	-0.281279
H	8.581894	-5.397824	0.082568
H	7.010362	-6.165644	0.329872
H	7.582851	-5.806744	-1.310246
C	6.947137	-0.835304	-2.221124
H	6.047001	-0.498200	-2.745855
H	7.434203	0.059470	-1.822826
H	7.613119	-1.283581	-2.963148

2 Anion

C	1.117731	0.776031	0.270963
C	1.264439	-0.567947	-0.204118
C	2.642015	-0.939140	-0.314507
C	3.536319	0.043146	0.034866
C	-0.130873	1.348226	0.476175
C	0.129929	-1.345811	-0.486289
C	-1.118679	-0.773597	-0.281130
C	-1.265390	0.570206	0.194435
H	-0.239902	2.365805	0.836855
H	0.238962	-2.363467	-0.846750
C	-2.642989	0.940699	0.306760

H	-2.969701	1.918696	0.642275
C	-3.537332	-0.041840	-0.041898
S	-2.687358	-1.519840	-0.544111
S	2.686441	1.522002	0.534674
H	2.968698	-1.917648	-0.648564
B	-5.085720	0.042617	-0.003397
C	-5.927218	-1.290474	-0.041259
C	-6.842945	-1.533663	-1.102614
C	-5.777875	-2.287853	0.953068
C	-7.545679	-2.737903	-1.157048
C	-6.512223	-3.479241	0.870466
C	-7.394879	-3.729297	-0.178606
H	-8.231526	-2.912113	-1.983658
H	-6.391277	-4.225066	1.653824
C	-5.737584	1.476688	0.062270
C	-6.618335	1.815311	1.125194
C	-5.465690	2.460727	-0.920588
C	-7.173078	3.095033	1.194284
C	-6.062573	3.724891	-0.830676
C	-6.909663	4.068267	0.223597
H	-7.835084	3.340388	2.022089
H	-5.860055	4.458522	-1.608523
C	-6.953263	0.823158	2.219448
H	-6.054088	0.481620	2.742904
H	-7.618809	1.268643	2.963550
H	-7.441659	-0.068554	1.815991
C	-7.510628	5.449013	0.329526
H	-8.528694	5.411828	0.729845
H	-6.921685	6.081017	1.005865
H	-7.541031	5.949638	-0.642443
C	-4.562485	2.204841	-2.113472
H	-4.944223	2.719768	-3.000481
H	-3.549667	2.579809	-1.927963
H	-4.466061	1.145821	-2.356374
C	-4.910985	-2.097973	2.181881
H	-4.282112	-2.976708	2.357948
H	-4.257537	-1.227443	2.113927
H	-5.539431	-1.967879	3.071012
C	-8.155118	-5.029872	-0.276935
H	-7.709840	-5.687699	-1.033408
H	-8.152149	-5.570348	0.673632
H	-9.195772	-4.861187	-0.572791
C	-7.048569	-0.526013	-2.214133
H	-7.319174	0.457916	-1.821882
H	-6.136435	-0.392339	-2.806857
H	-7.837196	-0.850696	-2.898019
B	5.084716	-0.042723	0.000783
C	5.927420	1.289604	0.037656
C	6.840019	1.534861	1.101231
C	5.781755	2.284620	-0.959642
C	7.543590	2.738664	1.154649
C	6.516913	3.475564	-0.877933
C	7.396645	3.727580	0.173133
H	8.226971	2.914520	1.982949
H	6.398893	4.219524	-1.663504
C	5.735841	-1.477445	-0.058338
C	5.459594	-2.458371	0.926140

C	6.615112	-1.821752	-1.120822
C	6.052005	-3.724967	0.839118
C	7.165443	-3.103399	-1.186880
C	6.902234	-4.071903	-0.211205
H	5.841487	-4.458412	1.615080
H	7.821237	-3.355285	-2.017693
C	7.041044	0.530221	2.216317
H	7.827638	0.856216	2.901917
H	6.126783	0.399064	2.806337
H	7.312093	-0.455074	1.827845
C	8.157593	5.027822	0.270381
H	7.709607	5.688571	1.022701
H	9.196813	4.859257	0.571278
H	8.159167	5.565117	-0.681994
C	4.918203	2.092264	-2.190395
H	4.293787	2.972828	-2.373005
H	5.549033	1.954890	-3.076741
H	4.260808	1.224888	-2.120243
C	4.551688	-2.199006	2.114645
H	4.453994	-1.139157	2.353402
H	3.539740	-2.575086	1.926628
H	4.930357	-2.710604	3.004891
C	7.547601	-5.434854	-0.281279
H	8.581894	-5.397824	0.082568
H	7.010362	-6.165644	0.329872
H	7.582851	-5.806744	-1.310246
C	6.947137	-0.835304	-2.221124
H	6.047001	-0.498200	-2.745855
H	7.434203	0.059470	-1.822826
H	7.613119	-1.283581	-2.963148

3

S	-3.6271888140	0.3843321887	2.4654784812
C	-3.8046122349	1.7188681411	1.3139430088
C	-2.6655308434	2.4969927474	1.3037574510
C	-1.6337909543	2.0543320033	2.1853370885
C	-2.0100077536	0.8885329567	2.8972658543
C	-0.3346675721	2.5891216003	2.4006473098
C	0.5107606454	1.9278313556	3.3145228819
C	0.1179854458	0.7697539218	4.0163923081
C	-1.1469426974	0.2458548562	3.8058920234
S	2.0651935812	2.7379280603	3.4154088439
C	1.5581453652	3.9316637595	2.2399581393
C	0.2868924840	3.7324573952	1.7973413023
B	-5.0927533850	1.8884922977	0.4688010052
H	-2.5772708141	3.3623861038	0.6570817573
H	-1.4677819566	-0.6476745155	4.3314014552
H	-0.2022284562	4.3642277643	1.0649755689
C	-5.0135243427	2.8218469653	-0.8002579141
C	-5.8755890565	3.9441381325	-0.9231349547
C	-6.9102607933	4.2637795692	0.1359898241
C	-5.7817165696	4.7802820089	-2.0371893093
H	-6.4387531011	5.6447029772	-2.1080623038
C	-4.8788024596	4.5260036570	-3.0773240913
C	-4.8343708685	5.4206260799	-4.2926042002
C	-4.0526090811	3.4080284612	-2.9650001930
H	-3.3577317564	3.1784521766	-3.7705474686

C	-4.0974604456	2.5626691107	-1.8479427064
C	-3.1668611881	1.3637467562	-1.8449469368
C	-6.4176854946	1.1363928964	0.8851787833
C	-7.0042385945	1.3280939778	2.1627850013
C	-6.4535923489	2.3027194923	3.1860739649
C	-8.1771842179	0.6477105954	2.5105487352
H	-8.6166755703	0.8261402318	3.4904675497
C	-8.8022041970	-0.2398987257	1.6333951712
C	-10.0671770088	-0.9627824270	2.0300837539
C	-8.2244392596	-0.4258621971	0.3734844538
H	-8.6964553953	-1.1119801717	-0.3266390580
C	-7.0639562116	0.2478853734	-0.0174899526
C	-6.5176499293	-0.0255757348	-1.4038354473
H	-7.3975255656	5.2207888628	-0.0692866144
H	-6.4711817923	4.3193368495	1.1365964543
H	-7.6845509250	3.4903508498	0.1748440525
H	-3.9872304238	5.1776933235	-4.9402422936
H	-4.7560494823	6.4753494883	-4.0072602013
H	-5.7500976834	5.3183419284	-4.8878056783
H	-3.0622728652	0.9597114390	-2.8569817999
H	-3.5155967538	0.5580808257	-1.1972267276
H	-2.1652977785	1.6396901013	-1.4970843457
H	-6.1240810905	1.7747520929	4.0875554986
H	-7.2317922062	3.0114627089	3.4900987336
H	-5.6050823066	2.8765270097	2.8143338391
H	-10.4257527715	-1.6122521472	1.2265835947
H	-10.8665759628	-0.2538306915	2.2744855352
H	-9.9062875135	-1.5820450055	2.9200248132
H	-7.1566023736	-0.7310575688	-1.9420189305
H	-5.5120085062	-0.4564785191	-1.3635904922
H	-6.4480549581	0.8901392640	-1.9971229184
H	0.7990964436	0.2896012782	4.7111775588
H	2.2471103657	4.7135780682	1.9495627417

3 Cation

S	-3.5651944560	0.2701576522	2.2080988075
C	-3.8136890293	1.7102912317	1.2832509808
C	-2.7148413272	2.5821718746	1.3796387661
C	-1.6730434771	2.0936886841	2.1829116198
C	-1.9704403468	0.8056606056	2.7199042793
C	-0.3980110684	2.6725191314	2.4882403148
C	0.4966571668	1.9219035627	3.3032520311
C	0.1819980928	0.6704150423	3.8086968406
C	-1.0800096123	0.0974908523	3.5087708700
S	2.0047541654	2.8027313170	3.5399902847
C	1.4210229699	4.1039138569	2.5768217524
C	0.1413821580	3.9079639597	2.0847515105
B	-5.1268444422	1.9041340462	0.4343962807
H	-2.6974627077	3.5278373993	0.8516375377
H	-1.3307037227	-0.8845044932	3.8953596952
H	-0.3754640581	4.6270560213	1.4613702278
C	-5.0051095502	2.7807662706	-0.8544416095
C	-5.8889967082	3.8759874020	-1.0489846935
C	-6.9856226406	4.2027796761	-0.0558535215
C	-5.7537100508	4.6811081301	-2.1809038287
H	-6.4203011163	5.5312031412	-2.3083505879
C	-4.7983502001	4.4127276117	-3.1688999360

C	-4.7132296676	5.2764197347	-4.4029796469
C	-3.9567102157	3.3124209360	-2.9889713229
H	-3.2256429639	3.0728631230	-3.7579446309
C	-4.0359784209	2.5027699867	-1.8510849084
C	-3.1012024368	1.3120888789	-1.7573645939
C	-6.4441523619	1.1995868096	0.8986848875
C	-6.9142412253	1.2987201447	2.2371753942
C	-6.2857113767	2.2206909264	3.2627147026
C	-8.0519027008	0.5929415391	2.6373280526
H	-8.4007495877	0.6934326546	3.6631731474
C	-8.7620082199	-0.2194600762	1.7504261516
C	-9.9920606744	-0.9669411027	2.2012360790
C	-8.3145374492	-0.2939820860	0.4265062434
H	-8.8608340477	-0.9166840667	-0.2781070102
C	-7.1948191282	0.4093730758	-0.0210886533
C	-6.8186453921	0.2740437722	-1.4826806150
H	-7.4561514441	5.1585939037	-0.3005749252
H	-6.6122855382	4.2643602712	0.9710933146
H	-7.7622507606	3.4306824408	-0.0642731679
H	-3.8620326269	4.9996910028	-5.0307206568
H	-4.6160450471	6.3350187396	-4.1382810270
H	-5.6233261530	5.1813629962	-5.0073657534
H	-2.7382038724	1.0277792440	-2.7490359795
H	-3.5828382217	0.4334985924	-1.3188898900
H	-2.2239927907	1.5362407794	-1.1405725904
H	-5.9015054276	1.6566956026	4.1192968413
H	-7.0392740643	2.9179785802	3.6447435093
H	-5.4683756759	2.8181943064	2.8575716021
H	-10.3559722736	-1.6461291887	1.4255350683
H	-10.8027462952	-0.2708597978	2.4472434770
H	-9.7885804599	-1.5527629570	3.1042098578
H	-7.4614888012	-0.4597355195	-1.9759458860
H	-5.7826903166	-0.0499400951	-1.6171504655
H	-6.9257565359	1.2256084917	-2.0118298530
H	0.8845983902	0.1205751146	4.4253099138
H	2.0507537449	4.9688202671	2.4108357119

3 Anion

S	-3.6331354583	0.4774843078	2.6191426523
C	-3.7757640252	1.7499213143	1.3414942788
C	-2.5541952731	2.4537600854	1.2990187613
C	-1.5532834475	2.0232218217	2.1969271462
C	-1.9765186706	0.9373609933	3.0227414601
C	-0.2190189543	2.5021273288	2.3850667994
C	0.5917990299	1.8867717521	3.3670644498
C	0.1503453653	0.8253383233	4.1735248402
C	-1.1563914388	0.3532304962	3.9883635363
S	2.1889433825	2.6380198118	3.3987279638
C	1.7434316249	3.7419536299	2.1042881213
C	0.4629130539	3.5514343974	1.6835308110
B	-5.0332943484	1.8988393345	0.5160625527
H	-2.4209982262	3.2792989210	0.6080274935
H	-1.5264352992	-0.4694274708	4.5940566360
H	0.0042469049	4.1319458468	0.8899952939
C	-5.0077432267	2.8506145719	-0.7659976856
C	-5.9268055777	3.9303759398	-0.8978925942
C	-6.9473404871	4.2239709452	0.1816918097

C	-5.9160678412	4.7527587329	-2.0285984574
H	-6.6242051736	5.5780023398	-2.0919144451
C	-5.0246754763	4.5430089037	-3.0872728460
C	-5.0570155871	5.4208649682	-4.3166348154
C	-4.1311763207	3.4782810443	-2.9724025768
H	-3.4384628541	3.2821772081	-3.7897125712
C	-4.1074414190	2.6408942084	-1.8462303016
C	-3.1095042624	1.5009561916	-1.8532506203
C	-6.3806478659	1.1299531667	0.9064726560
C	-7.0415635352	1.3356992818	2.1488997302
C	-6.5190312531	2.2927499272	3.2022343395
C	-8.2429839387	0.6768622299	2.4445324728
H	-8.7273600735	0.8714465381	3.4011416617
C	-8.8448220632	-0.2055553634	1.5457420060
C	-10.1441422477	-0.9003377705	1.8823742395
C	-8.2010325500	-0.4140287070	0.3223703026
H	-8.6457139625	-1.1005641026	-0.3967021324
C	-7.0026444807	0.2304272102	-0.0048857768
C	-6.3768156307	-0.0847475516	-1.3472970705
H	-7.4556280051	5.1759445531	-0.0010983700
H	-6.4853096769	4.2672808718	1.1721312750
H	-7.7089888045	3.4383319492	0.2332684955
H	-4.1964963763	5.2338299424	-4.9662687561
H	-5.0559483115	6.4841572427	-4.0508951424
H	-5.9631662940	5.2440215022	-4.9104443500
H	-2.8188291919	1.2419237757	-2.8767413585
H	-3.5146438756	0.6094655717	-1.3693859630
H	-2.1985281548	1.7583889539	-1.3016069286
H	-6.1726509511	1.7494938980	4.0890532960
H	-7.3103726178	2.9770923391	3.5300076227
H	-5.6788739314	2.8798310288	2.8315845624
H	-10.4905679067	-1.5226377420	1.0517454440
H	-10.9355904532	-0.1771493753	2.1126882830
H	-10.0376935114	-1.5454379517	2.7627844353
H	-6.9750085889	-0.8139042289	-1.9028203082
H	-5.3707725013	-0.4999246543	-1.2241978481
H	-6.2673729510	0.8102378231	-1.9669339003
H	0.8004990448	0.3763623821	4.9165780587
H	2.4679246662	4.4595613123	1.7437313312

4

C	1.38712	-2.90856	-0.68083
C	0.71182	-1.70947	-0.35307
C	1.61328	-0.63979	-0.09059
C	2.94598	-0.97358	-0.19894
C	0.69258	-4.09345	-1.00644
C	-0.71183	-1.70947	-0.35309
C	-1.38713	-2.90856	-0.68086
H	1.23691	-4.99814	-1.25566
S	-3.12083	-2.68085	-0.63655
S	3.12082	-2.68088	-0.63645
H	1.29913	0.36432	0.16929
C	-1.61329	-0.63978	-0.09061
H	-1.29914	0.36433	0.16928
C	-2.94599	-0.97357	-0.19897
C	-0.69258	-4.09344	-1.00646
H	-1.23692	-4.99813	-1.25569

B	4.16206	-0.03842	0.01031
C	3.88091	1.51462	0.01921
C	3.24553	2.16225	-1.07005
C	4.24256	2.30034	1.14719
C	2.98752	3.53820	-1.01404
C	3.94714	3.66508	1.17492
C	3.31647	4.30681	0.10284
H	2.51742	4.02018	-1.86910
H	4.21952	4.24705	2.05300
C	5.59598	-0.66909	0.19099
C	6.63298	-0.37274	-0.73621
C	5.88346	-1.56439	1.25028
C	7.88373	-0.97741	-0.60333
C	7.15675	-2.14101	1.35775
C	8.16862	-1.86951	0.43848
H	8.66081	-0.74828	-1.32978
H	7.35885	-2.81482	2.18831
C	6.40212	0.56209	-1.90481
H	7.32800	0.73332	-2.46018
H	5.67017	0.14923	-2.60843
H	6.01524	1.53057	-1.57655
C	9.52422	-2.52555	0.54323
H	9.60444	-3.37229	-0.14973
H	10.32659	-1.82576	0.28834
H	9.70910	-2.90759	1.55116
C	4.89248	-1.87465	2.35482
H	4.83036	-2.95283	2.53506
H	5.21548	-1.40917	3.29358
H	3.88620	-1.51341	2.14023
C	4.91711	1.68345	2.35466
H	4.29417	0.90579	2.80919
H	5.86615	1.21112	2.08594
H	5.11584	2.43679	3.12146
C	2.98665	5.77855	0.16678
H	2.04870	5.94530	0.71110
H	3.76627	6.34089	0.69022
H	2.86546	6.20693	-0.83229
C	2.84075	1.43708	-2.34092
H	3.37781	0.49935	-2.48745
H	1.77238	1.19234	-2.33139
H	3.02218	2.06925	-3.21576
B	-4.16207	-0.03841	0.01028
C	-3.88092	1.51463	0.01920
C	-3.24555	2.16227	-1.07005
C	-4.24255	2.30034	1.14720
C	-2.98753	3.53822	-1.01403
C	-3.94712	3.66508	1.17494
C	-3.31647	4.30682	0.10286
H	-2.51744	4.02021	-1.86909
H	-4.21948	4.24703	2.05303
C	-5.59598	-0.66909	0.19099
C	-6.63300	-0.37273	-0.73619
C	-5.88344	-1.56441	1.25027
C	-7.88375	-0.97740	-0.60329
C	-7.15673	-2.14103	1.35776
C	-8.16861	-1.86952	0.43851
H	-8.66084	-0.74827	-1.32973

H	-7.35881	-2.81485	2.18830
C	-4.89243	-1.87467	2.35479
H	-3.88616	-1.51345	2.14017
H	-5.21541	-1.40919	3.29355
H	-4.83033	-2.95286	2.53503
C	-9.52421	-2.52557	0.54328
H	-10.32659	-1.82579	0.28837
H	-9.60443	-3.37232	-0.14967
H	-9.70908	-2.90760	1.55121
C	-6.40217	0.56212	-1.90478
H	-6.01531	1.53061	-1.57651
H	-5.67022	0.14928	-2.60841
H	-7.32806	0.73334	-2.46014
C	-4.91707	1.68344	2.35468
H	-5.86614	1.21114	2.08598
H	-4.29414	0.90575	2.80917
H	-5.11576	2.43677	3.12150
C	-2.98663	5.77856	0.16682
H	-2.04863	5.94528	0.71104
H	-2.86553	6.20696	-0.83226
H	-3.76619	6.34088	0.69034
C	-2.84077	1.43711	-2.34093
H	-3.02218	2.06930	-3.21576
H	-1.77240	1.19235	-2.33140
H	-3.37785	0.49940	-2.48748

4 Cation

C	1.39034	-3.08676	-0.58441
C	0.71581	-1.93426	-0.09614
C	1.57436	-0.86435	0.20995
C	2.92208	-1.10243	-0.03286
C	0.70926	-4.20640	-1.01977
C	-0.71110	-1.93209	-0.09439
C	-1.39076	-3.07868	-0.58878
H	1.23584	-5.07759	-1.38602
S	-3.18885	-2.77867	-0.63307
S	3.19024	-2.79523	-0.62896
H	1.23628	0.10221	0.55453
C	-1.56599	-0.86496	0.22983
H	-1.22503	0.09416	0.59208
C	-2.91611	-1.09645	-0.00940
C	-0.71463	-4.20180	-1.02337
H	-1.24494	-5.06965	-1.39214
B	4.07157	-0.05279	0.03019
C	3.61264	1.42828	-0.14073
C	2.63856	1.81434	-1.10564
C	4.08032	2.42062	0.77709
C	2.10213	3.10647	-1.08549
C	3.50922	3.69389	0.77315
C	2.50581	4.04992	-0.13868
H	1.35942	3.38135	-1.82855
H	3.85116	4.43010	1.49372
C	5.52846	-0.54248	0.24221
C	6.53994	-0.16435	-0.68548
C	5.88197	-1.39330	1.32877
C	7.83273	-0.67154	-0.54611
C	7.19331	-1.86060	1.44952

C	8.17723	-1.52189	0.51442
H	8.59121	-0.39894	-1.27319
H	7.45670	-2.49288	2.29130
C	6.23012	0.76510	-1.85134
H	7.10683	0.87573	-2.49580
H	5.40454	0.37955	-2.46060
H	5.93530	1.75450	-1.48759
C	9.58372	-2.07560	0.63048
H	9.68028	-2.98696	0.02573
H	10.32169	-1.35322	0.26684
H	9.82211	-2.33231	1.66719
C	4.88431	-1.70766	2.43352
H	5.29087	-2.45084	3.12519
H	4.65505	-0.80045	3.00580
H	3.94313	-2.08996	2.03050
C	5.17635	2.12471	1.79223
H	4.89307	1.30652	2.46237
H	6.10257	1.83246	1.28939
H	5.37355	3.00962	2.40410
C	1.88068	5.42988	-0.08887
H	1.21490	5.51072	0.77936
H	2.64924	6.20476	0.00654
H	1.28866	5.62899	-0.98696
C	2.18345	0.90284	-2.24252
H	2.78520	-0.00430	-2.31989
H	1.13587	0.60748	-2.11299
H	2.26200	1.44160	-3.19365
B	-4.06060	-0.04428	0.09018
C	-3.59336	1.44304	0.01679
C	-2.63512	1.89494	-0.93383
C	-4.05510	2.37061	1.00143
C	-2.11501	3.19108	-0.84578
C	-3.50186	3.65013	1.06390
C	-2.51967	4.07376	0.15775
H	-1.38531	3.51841	-1.58048
H	-3.84158	4.33735	1.83236
C	-5.52580	-0.53214	0.23498
C	-6.51286	-0.06917	-0.68539
C	-5.91337	-1.47425	1.23074
C	-7.80471	-0.59058	-0.63481
C	-7.22526	-1.95939	1.26085
C	-8.17821	-1.54038	0.32917
H	-8.54227	-0.25480	-1.35698
H	-7.51114	-2.66740	2.03202
C	-4.97472	-1.86152	2.36315
H	-3.94614	-1.99352	2.02041
H	-4.96956	-1.06911	3.12249
H	-5.30304	-2.78805	2.84291
C	-9.58946	-2.09335	0.34520
H	-10.32475	-1.28441	0.26908
H	-9.74321	-2.76372	-0.51011
H	-9.78266	-2.65823	1.26165
C	-6.17645	0.96582	-1.75067
H	-5.88080	1.91242	-1.28819
H	-5.34417	0.63523	-2.38253
H	-7.04216	1.14550	-2.39454
C	-5.12813	1.99438	2.01472

H	-6.07100	1.75972	1.51188
H	-4.83908	1.11490	2.59924
H	-5.29970	2.82128	2.70993
C	-1.91501	5.45897	0.27454
H	-1.20277	5.49042	1.10896
H	-1.37678	5.73361	-0.63724
H	-2.68908	6.20938	0.46837
C	-2.17965	1.05158	-2.12218
H	-2.24938	1.64641	-3.03986
H	-1.13457	0.74170	-2.00610
H	-2.78609	0.15409	-2.25652

4 Anion

C	1.391426	-3.003974	-0.458313
C	0.718600	-1.759918	-0.241162
C	1.608515	-0.687707	-0.062369
C	2.970376	-1.000890	-0.123582
C	0.705510	-4.197727	-0.667002
C	-0.718603	-1.759918	-0.241172
C	-1.391426	-3.003974	-0.458340
H	1.244802	-5.125914	-0.828304
S	-3.137592	-2.763374	-0.423908
S	3.137591	-2.763375	-0.423833
H	1.283746	0.331294	0.118852
C	-1.608522	-0.687709	-0.062386
H	-1.283757	0.331292	0.118841
C	-2.970382	-1.000899	-0.123588
C	-0.705506	-4.197727	-0.667017
H	-1.244795	-5.125913	-0.828337
B	4.158632	-0.069538	0.009207
C	3.880798	1.498347	0.040566
C	3.215085	2.166133	-1.019855
C	4.295649	2.286404	1.149980
C	2.982674	3.548459	-0.954547
C	4.036423	3.658882	1.186815
C	3.375929	4.314925	0.141561
H	2.482071	4.037097	-1.789015
H	4.357865	4.235231	2.052760
C	5.632153	-0.663600	0.101640
C	6.594874	-0.377183	-0.905819
C	6.035067	-1.504263	1.169010
C	7.880594	-0.918127	-0.835389
C	7.337063	-2.026656	1.210921
C	8.275843	-1.751532	0.218651
H	8.595932	-0.687920	-1.623241
H	7.620879	-2.661403	2.049127
C	6.242671	0.485708	-2.099215
H	7.119488	0.672198	-2.726016
H	5.485303	0.002265	-2.727217
H	5.826595	1.449719	-1.793737
C	9.664509	-2.344543	0.257764
H	9.751875	-3.194646	-0.430579
H	10.420862	-1.611853	-0.043095
H	9.919097	-2.705959	1.258423
C	5.129749	-1.834734	2.339637
H	5.093631	-2.915606	2.514220
H	5.509020	-1.372853	3.259354

H	4.109142	-1.485215	2.185408
C	4.998252	1.659419	2.335926
H	4.379588	0.885142	2.801609
H	5.932654	1.173503	2.040469
H	5.228241	2.407086	3.100475
C	3.085648	5.795369	0.215001
H	2.219339	5.997207	0.857626
H	3.931338	6.348877	0.636632
H	2.864339	6.210963	-0.772494
C	2.735500	1.451796	-2.269769
H	3.262202	0.512631	-2.440212
H	1.669627	1.204111	-2.199791
H	2.864505	2.087866	-3.151812
B	-4.158636	-0.069545	0.009214
C	-3.880791	1.498337	0.040675
C	-3.215081	2.166192	-1.019705
C	-4.295622	2.286318	1.150150
C	-2.982666	3.548513	-0.954304
C	-4.036391	3.658793	1.187078
C	-3.375910	4.314905	0.141860
H	-2.482068	4.037206	-1.788742
H	-4.357814	4.235082	2.053070
C	-5.632164	-0.663598	0.101591
C	-6.594870	-0.377100	-0.905856
C	-6.035100	-1.504316	1.168910
C	-7.880600	-0.918029	-0.835471
C	-7.337104	-2.026694	1.210775
C	-8.275870	-1.751497	0.218511
H	-8.595928	-0.687758	-1.623313
H	-7.620938	-2.661485	2.048941
C	-5.129808	-1.834842	2.339540
H	-4.109176	-1.485392	2.185313
H	-5.509049	-1.372925	3.259252
H	-5.093768	-2.915714	2.514133
C	-9.664543	-2.344495	0.257572
H	-10.420880	-1.611796	-0.043304
H	-9.751893	-3.194591	-0.430781
H	-9.919167	-2.705919	1.258219
C	-6.242645	0.485881	-2.099180
H	-5.826591	1.449877	-1.793619
H	-5.485250	0.002497	-2.727193
H	-7.119446	0.672404	-2.725993
C	-4.998198	1.659249	2.336069
H	-5.932616	1.173371	2.040600
H	-4.379529	0.884924	2.801668
H	-5.228152	2.406857	3.100685
C	-3.085624	5.795343	0.215396
H	-2.219315	5.997138	0.858036
H	-2.864312	6.211001	-0.772071
H	-3.931313	6.348827	0.637061
C	-2.735493	1.451936	-2.269663
H	-2.864439	2.088083	-3.151659
H	-1.669634	1.204192	-2.199671
H	-3.262234	0.512810	-2.440199
